# Superballistic flow of viscous electron fluid induced by edge magnetoplasmons in point contacts

**DOI:** 10.1038/s41467-026-73154-5

**Published:** 2026-06-06

**Authors:** Xinghao Wang, Wenfeng Zhang, Loren N. Pfeiffer, Kirk W. Baldwin, K. W. West, Rui-Rui Du

**Affiliations:** 1 International Center for Quantum Materials, School of Physics, Peking University, Beijing, China; 2 Department of Electrical Engineering, Princeton University, Princeton, NJ USA; 3 Hefei National Laboratory, Hefei, China

**Keywords:** Semiconductors, Electronic properties and materials, Electronic devices

## Abstract

In narrow conductors, electron-electron collisions can create a viscous fluid state, allowing conductivity beyond ballistic transport into the superballistic regime. Point contacts made from ultrahigh-mobility two-dimensional electron gas serve as a platform to study this effect. Under microwave irradiation, edge magnetoplasmons in point contacts are excited and strongly influence electron dynamics. This study uses photoconductivity signals - changes from microwave exposure - to investigate superballistic electron flow, confirmed by size-dependent photoconductivity, magnetic field effects, and microwave power analysis. At low magnetic fields, weak microwaves lead to positive photoconductivity due to superballistic flow, while strong radiation causes negative photoconductivity because of enhanced phonon scattering.

## Abstract (web summary)

Superballistic transport is a known hydrodynamic effect where viscous electrons can move collectively. Here, the authors provide evidence of superballistic electron flow under microwave irradiation in point contacts induced by edge magnetoplasmons.

## Introduction

Hydrodynamic charge transport in two-dimensional electron gas (2DEG) has attracted increasing interest since the viscous fluid state of electrons was reported^1–15^ in graphene and GaAs/AlGaAs quantum wells. In this regime, frequent electron–electron collisions drive collective motion, resulting in phenomena, such as negative resistance due to whirlpools^8–11^, Hall viscosity^12^, negative magnetoresistance during Poiseuille flow^15–18^, quantum-critical conductivity^19^, violation of the Wiedemann–Franz law^20^, hydrodynamic plasmons^21,22^, and superballistic flow through narrow constrictions^23,24^. These effects are strongly influenced by electronic temperature, which affects the rate of momentum-conserving collisions. Superballistic transport is a notable hydrodynamic effect^23,24^ where viscous electrons can move collectively, leading to conductance above Sharvin’s formula by minimizing momentum loss at point contact (PC) edges^25,26^. This mirrors the shift from Knudsen to Poiseuille flow in gases. Superballistic transport demonstrates how electron–electron interactions can push conductance beyond ballistic limits, positioning hydrodynamic electron devices as key platforms for studying quantum many-body effects and creating advanced technologies like nanoscale detectors and efficient electronics.

Recent studies have demonstrated that microwave (MW) radiation can facilitate hydrodynamic transport. For instance, hydrodynamic charge transport under MW irradiation was observed in ultrahigh-mobility GaAs/AlGaAs 2DEG^15^ by analyzing size-dependent MW-induced resistance oscillation^27–32^. A key role of irradiation is to thermally decouple electrons from the lattice. In this context, the viscous terahertz photoconductivity of hydrodynamic electrons in graphene at zero magnetic field has been reported ^33^.

The role of edge magnetoplasmons (EMPs) in electron viscous flow has not yet been studied. EMPs represent a unique collective mode of plasmons that propagate along sample edges. They exhibit a broad frequency range (from kHz to THz) and minimal damping compared to bulk magnetoplasmons. EMPs can be excited by applying an AC bias between contacts^34–39^ or by directly irradiating the sample surface with MWs^40–42^. Notably, interference between EMPs generated at different contacts under MW irradiation leads to B-periodic resistance oscillations, known as EMP-induced resistance oscillations (EIROs). The magnetoresistance influenced by EMPs is proportional to |1 + exp(i*q*Δ*L*) |^2^ refs. ^43,44^, where the EMP wave vector $q\propto \omega B/n$, $\omega$ is the MW frequency, $n$ is the carrier density, and $\Delta L$ is the difference in EMP propagation lengths. Since EMPs with the same phase are generated by long-wavelength MW radiation (larger than the device size) near each metallic contact, $\Delta L$ is determined by the distance between contacts. EIROs have been reported in normal Hall bars^40–42^ and can be significantly amplified in narrow PCs, with a giant EIRO exhibiting a relative amplitude of approximately 700% observed in a bridged-gate tunnel PC^45^. Despite the rich physics associated with EIROs, there is limited research on PCs in the open regime (where PC conductance ${G}_{{{\rm{PC}}}} > {{{\rm{e}}}}^{2}/{{\rm{h}}}$, as opposed to the tunneling regime), in which electrons flow ballistically through the channel.

In this work, we combine hydrodynamic transport with EMP physics and present transport measurements of PCs in the open regime under MW irradiation using high- and ultrahigh-mobility samples. The resistance of the PC is found to depend on the MW power ${P}_{{{\rm{MW}}}}$, exhibiting behavior reminiscent of superballistic transport in graphene^24^. We provide evidence for the existence of superballistic electron flow under MW irradiation by observing size- and MW-power-dependent positive photoconductivity (reduced resistance) induced by EMPs. The mechanism underlying superballistic transport can be interpreted as follows. MW irradiation is absorbed by the 2DEG, exciting EMPs at the sample boundaries, which in turn heats electrons in the PC region and raises the effective electron temperature, ${T}_{e}$, significantly above the lattice temperature, ${T}_{{{\rm{l}}}}$. Therefore, MW irradiation indirectly enhances the momentum-conserving electron–electron scattering rate, $1/{\tau }_{{{\rm{ee}}}}$, in the PC region via thermal excitation. When ${\tau }_{{ee}}$ becomes comparable to other scattering timescales, the system reaches the hydrodynamic regime. Our findings reveal that constructive interference of EMPs in the PC region promotes hydrodynamic transport near the PC, whereas destructive interference does not enhance PC conductance, leaving electrons to flow ballistically through the constriction. These observations not only highlight intriguing nonlinear transport physics in the mesoscopic regime but also underscore the practical potential of electron hydrodynamics for applications, such as electron thermometers and MW sensors.

## Results

**Fig. 1:**
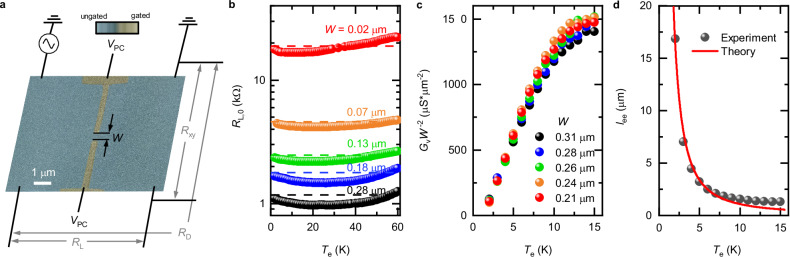
Superballistic transport in point contacts. **a** False-color scanning electron micrograph of PC A7, situated at the center of Hall bar (blue), schematically illustrated in Fig. 2b. As a representative split-gate PC, the device comprises two metallic top gates (yellow) that deplete the underlying electrons when a negative bias voltage ${V}_{{{\rm{PC}}}}$ is applied. For wet-etched PCs, the structure is similar, except that the top gates are replaced by etched regions of identical geometry. **b** The non-monotonic temperature dependence of ${R}_{{{\rm{L}}},0}$ for PC B2 at zero magnetic field illustrates the transition from ballistic to superballistic, followed by a regime dominated by phonon scattering. The PC width $W$ is tuned via ${V}_{{{\rm{PC}}}}$. Dash lines mark the ballistic PC resistance given by $1/{G}_{0}$. **c** Viscous conductance ${G}_{{{\rm{v}}}}$ normalized by ${W}^{2}$, measured in PC B2 for widths ranging from $0.21\,{{\rm{\mu }}}{{\rm{m}}}$ to $0.31\,{{\rm{\mu }}}{{\rm{m}}}$. **d** Electron–electron scattering length ${l}_{{{\rm{ee}}}}$, derived from the data in (**c**).

**Fig. 2:**
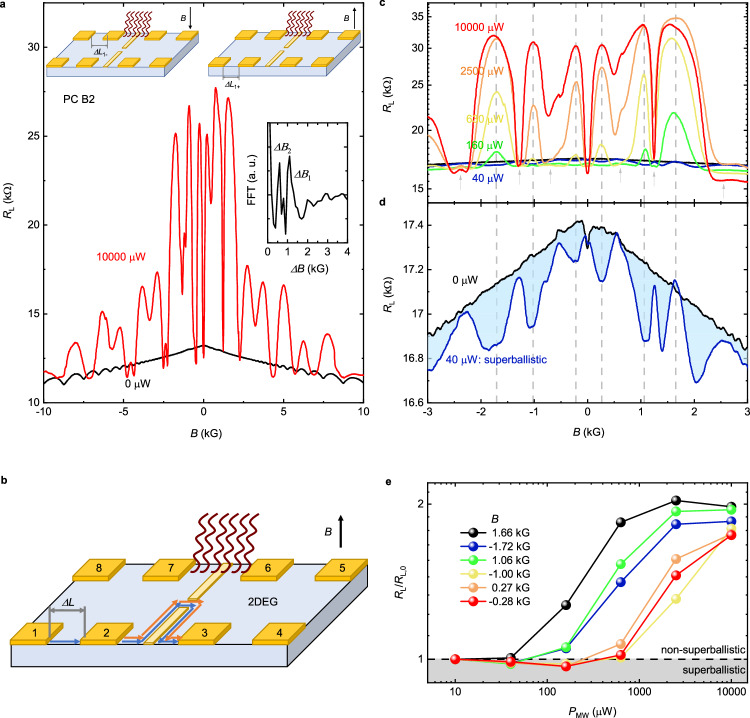
Resistance oscillation of point contacts induced by EMPs. **a** The longitudinal resistance ${R}_{{{\rm{L}}}}$ of PC B2 shows markedly different behavior with and without MW irradiation at a base temperature of 0.3 K and a MW frequency of 71 GHz. The observed asymmetry with respect to magnetic field $B$ arises from changes in the propagation length difference $\Delta L$ when EMPs reverse direction, denoted as $\Delta {L}_{1-}$ and $\Delta {L}_{1+}$ in the upper insets. The lower inset presents the Fourier transform of the red trace, revealing two distinct sets of EIROs. **b** Schematic of the Hall bar device, showing the interfering EMP propagation paths indicated by orange and blue arrows. **c** Evolution of EIROs with increasing MW power, illustrating both constructive (dashed lines) and destructive (arrows) interference patterns of EMPs. **d** Under weak MW radiation, ${R}_{{{\rm{L}}}}$ exhibits resistance minima at magnetic fields corresponding to constructive interference, in contrast to the resistance peaks observed under strong irradiation. **e** As MW power is varied, the PC resistance initially decreases (superballistic regime) and subsequently increases (phonon-scattering regime), indicating a transition from ballistic to superballistic to phonon-scattering dominated transport.

Figure 1a shows a scanning electron micrograph of a split-gate PC. The split gates deplete the underlying electrons to form a narrow constriction, which can also be fabricated by etching. The longitudinal resistance ${R}_{{{\rm{L}}}}$ across the PC (e.g., between contacts “2” and “3” in Fig. 2b) is measured using a low-frequency (17 Hz) lock-in technique. Owing to the high mobility of our samples, the contribution of the bulk 2DEG to ${R}_{{{\rm{L}}}}$ is negligible at a bath temperature around $0.3{{\rm{K}}}$. According to the Landauer-Büttiker formula^46^, the longitudinal resistance in the absence of MW radiation is given by ${R}_{{{\rm{L}}},0}={R}_{{{\rm{D}}}}-\left|{R}_{{{\rm{xy}}}}\right|={{\rm{h}}}/2{{{\rm{e}}}}^{2}{N}_{{{\rm{PC}}}}-\left|{R}_{{{\rm{xy}}}}\right|$ (see black line in Fig. 2a), where the diagonal resistance ${R}_{{{\rm{D}}}}$ corresponds to the PC resistance and ${N}_{{{\rm{PC}}}}$ is the number of conducting channels in the PC. Here, ${R}_{{{\rm{L}}}}$ in the absence of MW radiation is denoted as ${R}_{{{\rm{L}}},0}$. For narrow PCs, we observe quantized resistance, indicating the quantum PC regime ^26^.

Superballistic transport was observed in our PCs by varying the lattice temperature $({T}_{{{\rm{l}}}})$ (Fig. 1b). The electron temperature $({T}_{{{\rm{e}}}})$ is equal to ${T}_{{{\rm{l}}}}$ in this case. At zero magnetic field, ${R}_{{{\rm{L}}},0}$ exhibits a non-monotonic temperature dependence, revealing a transition from ballistic to superballistic and finally to a phonon-scattering dominated regime—a progression widely studied in the graphene system^24^. In the superballistic regime, ${R}_{{{\rm{L}}},0}$ falls below the ballistic value because electron viscosity enhances the PC conductance from ${G}_{0}=2{{{\rm{e}}}}^{2}{N}_{{{\rm{PC}}}}/{{\rm{h}}}$ to ${G}_{0}+{G}_{{{\rm{v}}}}$ ^23,24^ :

1 $${G}_{{{\rm{v}}}}=\frac{ {\sqrt {2\pi n}}{e}^{2}{W}^{2}}{16 \hslash {{v}_{{{\rm{F}}}}} \tau_{ee}}$$

Where ${v}_{{{\rm{F}}}}$ is the Fermi velocity, ${\tau }_{{{\rm{ee}}}}$ is the viscous scattering time, and the electrical width $W$ can be estimated using Sharvin’s formula as $W={\pi {{\rm{h}}}}/(2{{{\rm{e}}}}^{2}\sqrt{2\pi n}R_{{{\rm{L}}},0})$, evaluated at zero magnetic field and a temperature of 0.3 K^25,26^. In the phonon-scattering dominated regime, ${R}_{{{\rm{L}}},0}$ exceeds the ballistic value due to enhanced scattering between electrons and phonons.

We measured the electron-temperature and size dependence of ${G}_{{{\rm{v}}}}$ (Fig. 1c). The viscosity conductance scales quadratically with $W$, a hallmark of Poiseuille-type flow in which the electron fluid self-organizes into streams with a parabolic velocity profile. From these measurements, we extracted the electron–electron scattering length ${l}_{{{\rm{ee}}}}={v}_{{{\rm{F}}}}{\tau }_{{{\rm{ee}}}}$, which theoretically follows ${{l}_{{{\rm{ee}}}}\propto T}_{{{\rm{e}}}}^{-2}{{\mathrm{ln}}}^{-1}({E}_{{{\rm{F}}}}/{{{\rm{k}}}}_{{{\rm{B}}}}{T}_{{{\rm{e}}}})$, where ${{{\rm{k}}}}_{{{\rm{B}}}}$ is the Boltzmann constant, and ${E}_{{{\rm{F}}}}$ is the Fermi energy (Fig. 1d). Above $10{{\rm{K}}}$, phonon scattering becomes dominant and strongly affects ${R}_{{{\rm{L}}},0}$, causing deviations of ${G}_{{{\rm{v}}}}$ from Eq. 1 and of the measured ${l}_{{{\rm{ee}}}}$ from the theoretical prediction. Although ${l}_{{{\rm{ee}}}}$ could still exceed $W$ around $10{{\rm{K}}}$, indicating that the PCs operate near the ballistic regime, superballistic transport remains clearly observable in our samples.

Under MW irradiation, ${R}_{{{\rm{L}}}}$ deviates from ${R}_{{{\rm{L}}},0}$ and exhibits magnetic-field-dependent oscillations. Since the Hall resistance ${R}_{{{\rm{xy}}}}$ of the 2DEG remains unaffected by MW radiation, the response of ${R}_{{{\rm{L}}}}$ to MW irradiation directly reflects the behaviors of the PCs. As illustrated in Fig. 2b, we observe EMP excitation in the PCs, giving rise to B-periodic EIROs, shown by the red curve in Fig. 2a. The oscillation period follows the relation $\Delta B\propto 1/\omega$ (See Supplementary Information (SI) for more details). Specifically, we identify two distinct sets of EIROs with periods $\Delta {B}_{1}=1.1{{\rm{kG}}}$ and $\Delta {B}_{2}=0.6{{\rm{kG}}}$ (lower inset of Fig. 2a). These correspond to two different EMP propagation length differences $\Delta {L}_{1}$ and $\Delta {L}_{2}$, associated with the nearest and second-nearest contact pairs along the sample edge. Furthermore, the observed asymmetry in $B$ can be attributed to variations in $\Delta L$ when EMPs reverse their propagation direction (denoted as $\Delta {L}_{1-}$ and $\Delta {L}_{1+}$ in the upper insets of Fig. 2a). The resistance maxima of EIROs in Fig. 2a occur when $q\Delta L/2{{\rm{\pi }}}$ is an integer.

The wave vector magnitude $q$, which scales linearly with magnetic field $B$, is determined from the EMP dispersion relation^45^: $q={{{\rm{\epsilon }}}}_{0}\left({\epsilon }_{{{\rm{r}}}}+1\right){m}^{*}\omega \left(0.80{\omega }_{{{\rm{c}}}}+1.22\omega \right)/n{e}^{2}$, where ${{{\rm{\epsilon }}}}_{0}$ is the vacuum permittivity, ${\epsilon }_{{{\rm{r}}}}=12.9$ is the relative permittivity of GaAs, ${m}^{*}=0.067{{{\rm{m}}}}_{{{\rm{e}}}}$ is the effective electron mass, and ${\omega }_{{{\rm{c}}}}={{\rm{e}}}B/{m}^{*}$ is the cyclotron frequency. With a dielectric thickness of approximately 200 nm between the 2DEG and the sample surface, the coefficients preceding ${\omega }_{{{\rm{c}}}}$ and $\omega$ in the dispersion relation have been calculated accordingly (see SI for more details). The two observed sets of EIROs correspond to propagation length differences of $\Delta {L}_{1}=530\,{{\rm{\mu }}}{{\rm{m}}}$ and $\Delta {L}_{2}=980\,{{\rm{\mu }}}{{\rm{m}}}$, which closely match the distances between the nearest contacts ($\sim 500\,{{\rm{\mu }}}{{\rm{m}}}$) and the second-nearest contacts ($\sim 1000\,{{\rm{\mu }}}{{\rm{m}}}$), respectively.

The dependence of EIROs on MW power ${P}_{{{\rm{MW}}}}$ is relatively complex (Fig. 2c). At high ${P}_{{{\rm{MW}}}}$, ${R}_{{{\rm{L}}}}$ (warm-colored traces) exceeds ${R}_{{{\rm{L}}},0}$ (black trace) under conditions of constructive EMP interference (dash lines), as the system enters the phonon-scattering dominated regime—a typical behavior of PCs in the open regime^47^. However, at low ${P}_{{{\rm{MW}}}}$ (e.g., $40\,{{\rm{\mu }}}{{\rm{W}}}$), ${R}_{{{\rm{L}}}}$ can drop below ${R}_{{{\rm{L}}},0}$, providing direct evidence of superballistic transport (shown separately in Fig. 2d for clarity). At magnetic fields corresponding to constructive EMP interference, the resistance minima evolve into maxima as ${P}_{{{\rm{MW}}}}$ increases (e.g., the right dash line in Fig. 2d). As shown in Fig. 2e, ${R}_{{{\rm{L}}}}$ in these fields consistently exhibits an initial decrease (superballistic regime) followed by a sharp increase (phonon-scattering dominated regime, also reported in ref. ^24^). The superballistic signature in Fig. 2d appears relatively subtle due to the limited number of conduction channels and near-ballistic character of this PC (although superballistic signature exists, the PC is not in the hydrodynamic regime). More pronounced examples will be presented later using other PCs. In contrast, at magnetic fields where destructive EMP interference occurs, ${R}_{{{\rm{L}}}}$ remains close to ${R}_{{{\rm{L}}},0}$ even under strong MW irradiation. These phenomena are consistently observed in both split-gate and etched PCs, as well as in high- and ultrahigh-mobility samples.

**Fig. 3:**
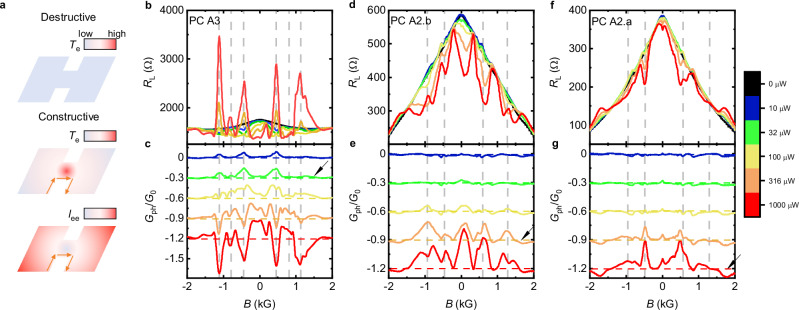
Longitudinal resistance *R*_L_ and normalized photoconductance *G*_ph_/*G*_o_ of point contacts with three different sizes. **a** Schematic diagram illustrating the mechanism of EIROs. Constructive interference of EMPs locally heats electrons in the PC region, significantly raising the electron temperature ${T}_{{{\rm{e}}}}$ above the bath temperature. This reduces the electron–electron scattering length and modifies transport properties. **b**, **c** Data for PC A3 ($W=0.18\,{{\rm{\mu }}}{{\rm{m}}}$) showing ${R}_{{{\rm{L}}}}$ and normalized photoconductance ${G}_{{{\rm{ph}}}}/{G}_{0}$ respectively. **d**, **e** Corresponding data for PC A2.b ($W=0.54\,{{\rm{\mu }}}{{\rm{m}}}$). **f**, **g** Data for PC A2.a ($W=0.82\,{{\rm{\mu }}}{{\rm{m}}}$). All measurements were performed at a base temperature of 0.3 K with an MW frequency of 71 GHz. Dashed lines mark magnetic field positions where superballistic transport emerges. The traces marked by arrows lie near the positive-to-negative photoconductance crossover and represent comparable ${P}_{{{\rm{a}}}}$ values. Consistent with the observations in Fig. 2a, the asymmetry of EIROs around *B *= 0 T persists, reflecting the dependence of EMP propagation length on magnetic field direction. For clarity, successive ${G}_{{{\rm{ph}}}}/{G}_{0}$ traces at different MW power levels are vertically offset by −0.3. The same color scale, shown on the right, applies to all data in (**a**) through (**g**).

Data demonstrating EMP-induced superballistic transport are presented in Fig. 3c (blue, green, and yellow traces), as well as in Fig. 3e, g. To quantitatively analyze this phenomenon, we introduce the photoconductance ${G}_{{{\rm{ph}}}}$, defined as ${G}_{{{\rm{ph}}}}=1/({R}_{{{\rm{L}}}}-{R}_{{{\rm{L}}},0}+ {R}_{{{\rm{L}}},0}(B=0))\,-1/{R}_{{{\rm{L}}},0}(B=0)$, which is analogous to the viscous conductance ${G}_{{{\rm{v}}}}$. This quantity, normalized by ${G}_{0}$ is plotted in Fig. 3b, d, f. In magnetic field regions with constructive EMP interference, the evolution of resistance minima into maxima corresponds to a transition in ${G}_{{{\rm{ph}}}}$ from positive to negative values.

We now turn to explain how EMPs cause superballistic transport. The 2DEG absorbs MW irradiation, generating EMPs at the sample boundaries. These EMPs dramatically heat the electrons within the PC region, raising their effective temperature, ${T}_{{{\rm{e}}}}$, far above the bath temperature (Fig. 3a). The absorbed MW power ${P}_{{{\rm{a}}}}$ for the bulk 2DEG is inversely proportional to its conductivity, *P*_a_ ∝ 1/*σ*^48^. Consequently, the PCs absorb significantly more power than the bulk, leading to localized heating. This explains why even weak MW radiation can heat the electrons in the narrow constrictions to ${T}_{{{\rm{e}}}}\gg 1\,{{\rm{K}}}$, driving them into the hydrodynamic regime, while the bulk 2DEG remains cold ($< 1{{\rm{K}}}$). To rule out global heating, we monitored Shubnikov-de Haas (SdH) oscillations in the bulk 2DEG under MW irradiation. Their persistence at low magnetic fields confirms that the global electron temperature stays well below 1 K (see “Methods”), a condition ensured by the cryostat’s cooling capacity and effective thermal anchoring.

The scaling ${P}_{{{\rm{a}}}}\propto 1/\sigma$ also holds for PCs of varying sizes. This is because the Joule heating from EMPs (photo-induced high-frequency current) in a PC is proportional to its resistance, leading to an effective absorbed power ${P}_{{{\rm{a}}}}\propto 1/W\propto 1/\sigma$. The size dependence of this effect is demonstrated by the EIROs in Fig. 3. Panels d–g show resistance traces for split-gate PCs at different gate voltages, corresponding to different widths (e.g., ${V}_{{{\rm{PC}}}}=-6.0\,{{\rm{V}}}$ for the narrower PC A2.b and ${V}_{{{\rm{PC}}}}=-2.2\,{{\rm{V}}}$ for the wider PC A2.a). At a fixed MW power ${P}_{{{\rm{MW}}}}$, the narrower PCs exhibit a significantly stronger response, consistent with a higher electron temperature ${T}_{{{\rm{e}}}}$ induced by more efficient EMP heating. In contrast, extremely wide PCs ($W > 2\,{{\rm{\mu }}}{{\rm{m}}}$) show no EMP signal, as EMP interference becomes negligible in such systems (see SI for more details).

Superballistic transport is suppressed by magnetic fields. The field bends electron trajectories, thereby restricting the momentum-conserving electron–electron scattering that is protected under time-reversal symmetry. To characterize the field strength, we introduce the dimensionless parameter $\beta=W/2{R}_{{{\rm{c}}}}$, where ${R}_{{{\rm{c}}}}={m}^{*}{v}_{{{\rm{F}}}}/{{\rm{e}}}B$ is the cyclotron radius. Within the regime ${{{\rm{k}}}}_{{{\rm{B}}}}^{2}{T}_{{{\rm{e}}}}^{2}/{E}_{{{\rm{F}}}}^{2}\, \ll \, \beta \, \ll \, 1$ (corresponding to magnetic fields of approximately $0.2\,{{\rm{kG}}}-2\,{{\rm{kG}}}$), the viscosity scattering rate is given by ref. ^49^

2 $$\frac{1}{{{\rm{T}}}_{{\mathrm{ee}}}}=\frac{64{\alpha }_{{\mathrm{ee}}}{{\rm{k}}}_{{\rm{B}}}^{2}{T}_{e}^{2}}{9{\pi }^{2} \hslash \sqrt{\beta }{E}_{{{\rm{F}}}}}{{\mathrm{ln}}}\, \left(\frac{\sqrt{\beta }{E}_{{{\rm{F}}}}}{{{{\rm{k}}}}_{{{\rm{B}}}}{T}_{{{\rm{e}}}}}\right),$$

where ${\alpha }_{{{\rm{ee}}}}$ is a dimensionless parameter characterizing electron–electron scattering strength.

**Fig. 4:**
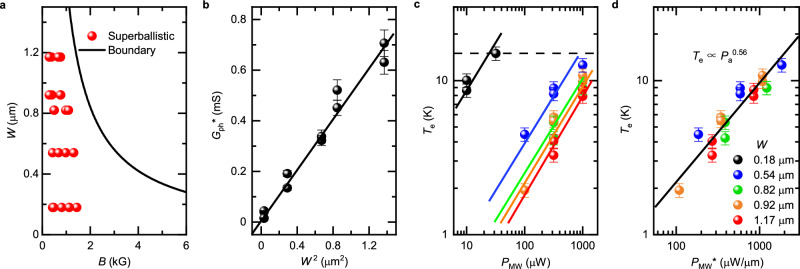
Evidence for superballistic transport induced by microwave irradiation. **a** Superballistic transport (red spheres) becomes suppressed in the non-hydrodynamic regime (above the solid boundary line). **b** The effective photoconductance ${G}_{{{\rm{ph}}}}^{*}$ exhibits a linear dependence on ${{{\rm{W}}}}^{2}$, consistent with the prediction of Eq. 1. The error bars represent the standard error (SE) of the effective photo**c**onductance. **c** The extracted electron temperature ${T}_{{{\rm{e}}}}$ for superballistic electrons under constructive interference conditions near $B=0.5\,{{\rm{kG}}}$ is shown for PCs of different sizes. A dashed line indicates the saturation of ${T}_{{{\rm{e}}}}$, attributed to the onset of electron-phonon scattering. The solid lines represent the best-fit curves for each PC, each constrained to be parallel to the fitting line in (**d**). **d** After renormalizing the applied MW power ${P}_{{{\rm{MW}}}}$ to the absorbed power ${P}_{{{\rm{MW}}}}^{*}$, we observe that ${T}_{{{\rm{e}}}}$ follows a power-law dependence on the MW power. The dominant uncertainty in ${T}_{{{\rm{e}}}}$ of (**c**, **d**) arises from the uncertainty in the electron–electron interaction parameter ${\alpha }_{{{\rm{ee}}}}$, shown as the error bars representing the SE. The solid line represents the best-fit line, yielding the relationship ${T}_{{{\rm{e}}}}\propto {{P}_{{{\rm{MW}}}}^{*}}^{0.56}$.

The positive photoconductance in narrower PCs exhibits greater resilience to magnetic fields. For instance, the EMP-induced positive photoconductance of the narrower PC A2.b near $B=\pm 1\,{{\rm{kG}}}$ is significantly stronger than that of the wider PC A2.a. This resilience stems from the suppression of the viscous scattering rate, $1/{\tau }_{{{\rm{ee}}}}$, by the dimensionless magnetic field parameter $\beta \propto {BW}$. As $\beta$ approaches 1, the magnetic field bends electron trajectories significantly, which strongly suppresses the momentum-conserving electron–electron scattering essential for superballistic transport. This transition is demarcated in Fig. 4a by a boundary line separating the regimes where superballistic transport is permitted ($\beta < 1$) and prohibited ($\beta > 1$).

The positive photoconductance under a magnetic field exhibits a size dependence consistent with hydrodynamic Poiseuille flow, where the viscous conductance scales as ${G}_{{{\rm{v}}}}\propto {W}^{2}$, similar to the superballistic transport at zero field. To demonstrate this scaling, we compare resistance traces for different PCs at similar absorbed power levels ${P}_{{{\rm{a}}}}$, which corresponds to similar electron temperature ${T}_{{{\rm{e}}}}$. For example, the traces marked by arrows in Fig. 3—achieved with ${P}_{{{\rm{MW}}}}=32\,{{\rm{\mu }}}{{\rm{W}}}$ for PC A3, $316\,{{\rm{\mu }}}{{\rm{W}}}$ for PC A2.b, and $1000\,{{\rm{\mu }}}{{\rm{W}}}$ for PC A2.a-all lie near the positive-to-negative photoconductance crossover and represent comparable ${P}_{{{\rm{a}}}}$ values. Under these conditions, the peaks of normalized photoconductance ${G}_{{{\rm{ph}}}}/{G}_{0}$ increase almost linearly with the PC width $W$. Since ${G}_{0}=2{{{\rm{e}}}}^{2}{N}_{{{\rm{PC}}}}/{{\rm{h}}}$ also scales linearly with $W$, the observed increase implies ${G}_{{{\rm{ph}}}}\propto {W}^{2}$. To account for the correction term in Eq. 2, we define an effective photoconductance ${G}_{{{\rm{ph}}}}^{*}={G}_{{{\rm{ph}}}}\sqrt{\beta }/{\mathrm{ln}}(\sqrt{\beta }{E}_{{{\rm{F}}}}/{{{\rm{k}}}}_{{{\rm{B}}}}{T}_{{{\rm{e}}}})$, with ${P}_{{{\rm{a}}}}$ (and thus ${T}_{{{\rm{e}}}}$) and $B$ held fixed. We evaluate this at $B\sim 0.5\,{{\rm{kG}}}$ (corresponding to the first EIRO maximum) and ${T}_{{{\rm{e}}}}\sim 10{{\rm{K}}}$ (extracted from Fig. 4c at the photoconductance crossover, see “Methods”). As shown in Fig. 4b, ${G}_{{{\rm{ph}}}}^{*}$ is indeed proportional to ${W}^{2}$. Given that the correction term exhibits negligible dependence on ${T}_{{{\rm{e}}}}$, this observed quadratic scaling provides robust and unambiguous evidence for superballistic transport in our system.

We now explain why the dependence of positive photoconductance ${G}_{{{\rm{ph}}}}$ on MW power mirrors the dependence of viscous conductance ${G}_{{{\rm{v}}}}$ on temperature in superballistic transport. The key lies in the electron heating dynamics: MW irradiation raises the effective electron temperature following ${T}_{{{\rm{e}}}}\propto {P}_{{{\rm{a}}}}^{1/\gamma }$, where the exponent $1/\gamma$ is determined by the dominant energy relaxation mechanism coupling the heated PC electrons to the cold bath. Consequently, the ${G}_{{{\rm{v}}}}\left({T}_{{{\rm{e}}}}\right)$ dependence directly translates to the observed ${G}_{{{\rm{ph}}}}\left({P}_{{{\rm{a}}}}\right)$ dependence.

At thermal equilibrium, the absorbed MW power ${P}_{{{\rm{a}}}}$ is balanced by the power ${P}_{{{\rm{tr}}}}$ transferred from the PC region to the cold bath. This heat flow typically follows a power-law relation: ${{P}_{{{\rm{a}}}}=P}_{{{\rm{tr}}}}=\varSigma V\left({T}_{{{\rm{e}}}}^{\gamma }-{T}_{{{\rm{b}}}}^{\gamma }\right),$ where ${T}_{{{\rm{b}}}}$ is the bath temperature, $\varSigma$ is the coupling constant, and $V$ is the effective volume of the heated region. Under significant MW heating (${T}_{{{\rm{e}}}}\gg {T}_{{{\rm{b}}}}$), the term ${T}_{{{\rm{b}}}}^{\gamma }$ is negligible. For narrow constrictions, the thermal conductance in the electrical thermal transport regime scales linearly with ${T}_{{{\rm{e}}}}$, corresponding to a heat flow with ${P}_{{{\rm{a}}}}\propto {T}_{{{\rm{e}}}}^{2}$^50^ (see SI for more details), which gives ${T}_{{{\rm{e}}}}\propto {P}_{{{\rm{a}}}}^{0.5}$ (the cold bath is the bulk 2DEG). However, if electron-phonon coupling (the bath is the sample lattice, i.e., ${T}_{{{\rm{b}}}}={T}_{{{\rm{l}}}}$) were the dominant relaxation channel, the exponent would deviate from $0.5$. In the electron-phonon coupling process, ${P}_{{{\rm{a}}}}\propto {T}_{{{\rm{e}}}}^{5}$, which would lead to a much weaker power dependence, ${T}_{{{\rm{e}}}}\propto {P}_{{{\rm{a}}}}^{0.2}$.

The electron temperature ${T}_{{{\rm{e}}}}$ in the constructive interference regime was extracted from the positive photoconductance ${G}_{{{\rm{ph}}}}$ using Eq. 2, with the interaction strength parameter ${\alpha }_{{{\rm{ee}}}}=0.5$ as estimated from ref. ^51^. To ensure the analysis reflects only the superballistic transport regime, we used data from the resistance minima and excluded the maxima, which are dominated by phonon scatterings. The resulting ${T}_{{{\rm{e}}}}$ values are plotted in Fig. 4c. Our analysis is limited by the lack of a direct measurement for ${T}_{{{\rm{e}}}}$, and therefore proceeds under the assumption of the hydrodynamic model. To account for the size-dependent MW absorption rate, which scales as $1/\sigma \propto 1/W$, we defined a unified absorbed power for each PC by scaling the incident power as ${P}_{{{\rm{a}}}}\sim {P}_{{{\rm{MW}}}}^{*}={P}_{{{\rm{MW}}}}/W$. Plotting the extracted ${T}_{{{\rm{e}}}}$ against this scaled power ${P}_{{{\rm{MW}}}}^{*}$ reveals a clear power-law relationship: ${T}_{{{\rm{e}}}}\propto {{P}_{{{\rm{MW}}}}^{*}}^{0.56}$ as shown in Fig. 4d. This exponent is close to the theoretical value of $1/\gamma=0.5$.

**Fig. 5:**
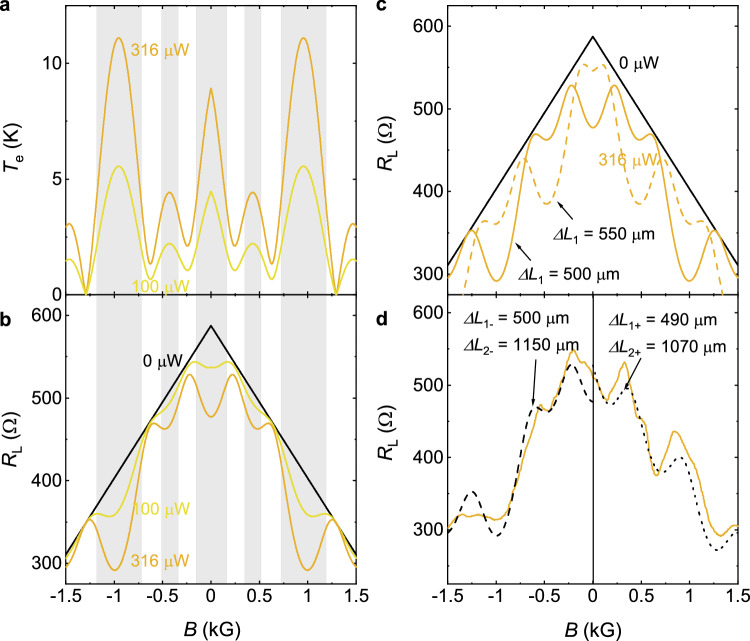
Simulation of electron temperature *T*_e_ and longitudinal resistance *R*_L_ according to the superballistic model. Calculated electron temperature ${T}_{{{\rm{e}}}}$ (**a**) and longitudinal resistance ${R}_{{{\rm{L}}}}$ (**b**) as functions of magnetic field under MW power ${P}_{{{\rm{MW}}}}=316\,{{\rm{\mu }}}{{\rm{W}}}$ and $100\,{{\rm{\mu }}}{{\rm{W}}}$, based on the hydrodynamic model. Simulation parameters are identical to those of PC A2.b in Fig. 3d. Gray-shaded regions indicate hydrodynamic transport regimes for the yellow and orange traces. **c** ${R}_{{{\rm{L}}}}$ traces show strong dependence on $\Delta {L}_{1}$, while $\Delta {L}_{2}$ is held fixed at $1150\,{{\rm{\mu }}}{{\rm{m}}}$. The orange solid (dashed) line represents $\Delta {L}_{1}=500\,{{\rm{\mu }}}{{\rm{m}}}$ ($\Delta {L}_{1}=550\,{{\rm{\mu }}}{{\rm{m}}}$) under MW power ${P}_{{{\rm{MW}}}}=316\,{{\rm{\mu }}}{{\rm{W}}}$. Here, $\Delta {L}_{2}$ determines the magnetic field positions of constructive interference, while $\Delta {L}_{1}$ sensitively mo**d**ulates the photoconductance amplitude. The black lines in (**b**, **c**) show the calculated ${R}_{{{\rm{L}}},0}$ without MW irradiation. **d** The orange trace from Fig. 3d is fitted separately for positive (dotted line) and negative (dashed line) magnetic fields using the theoretical hydrodynamic model. The discontinuity near zero field arises from limitations of the theoretical model in the low-field regime.

The longitudinal resistance ${R}_{{{\rm{L}}}}$ can be simulated based on the following physical picture: MW radiation generates EMPs, whose interference within the PC region governs the local electron temperature ${T}_{{{\rm{e}}}}$. As the magnetic field varies, the system oscillates between the superballistic and ballistic transport regimes due to the changing nature of this EMP interference. Figure 5 presents a simulation of ${T}_{{{\rm{e}}}}$ and ${R}_{{{\rm{L}}}}$ based on our combined EMP and hydrodynamic model, which shows excellent agreement with the experimental data. Figure 5a demonstrates the oscillatory behavior of ${T}_{{{\rm{e}}}}$, which results from the alternating constructive (heating) and destructive (cooling) interference of EMPs. Figure 5b shows the simulated EIROs for PC A2.b at two different MW powers ${P}_{{{\rm{MW}}}}$, successively reproducing the key features observed experimentally in Fig. 3c. Figure 5d provides an example of fitting the ${R}_{{{\rm{L}}}}$ trace directly using the hydrodynamic model. The details of these calculations are provided in the “Methods” section.

To further validate the hydrodynamic model under MW irradiation, we calculated the electron–electron scattering length ${l}_{{{\rm{ee}}}}$ in finite magnetic fields. The ratio ${l}_{{{\rm{ee}}}}/W$ provides a direct criterion for identifying the superballistic transport regime. For the cases exhibiting positive ${G}_{{{\rm{ph}}}}$, we find ${l}_{{{\rm{ee}}}}\sim W$ (see “Methods”), which is consistent with the data presented in Fig. 1d. Crucially, superballistic transport does not require the extreme hydrodynamic condition of ${l}_{{{\rm{ee}}}}\ll W$, and viscous effects can manifest even near the ballistic boundary where *l*_ee_ ≳ *W*^23^. Therefore, our experimental observation of ${l}_{{{\rm{ee}}}}\sim W$ is fully consistent with the occurrence of superballistic transport. We also compared the cyclotron radius ${R}_{c}$ with the characteristic viscous length scale *D*_v_ = $\sqrt {{{l}_{{\rm{ee}}}}{{l}_{0}}}/2$, where ${l}_{0}$ is the electron mean free path. Hydrodynamic transport is suppressed when ${D}_{{{\rm{v}}}}\gg {R}_{{{\rm{c}}}}$, as ballistic skipping orbitals begin to dominate. In our experiments, at $B=0.5\,{{\rm{kG}}}$, we have ${R}_{{{\rm{c}}}}=1.6\,{{\rm{\mu }}}{{\rm{m}}}$, while ${D}_{{{\rm{v}}}}$ is estimated to be smaller than $2\,{{\rm{\mu }}}{{\rm{m}}}$ (see “Methods”). This confirms that electrons are in or near the hydrodynamic regime at low fields. The subsequent diminishment of the hydrodynamic effect for $B > 1\,{{\rm{kG}}}$ aligns perfectly with this picture, as increasing $B$ further reduces ${R}_{{{\rm{c}}}}$, eventually leading to the dominance of ballistic transport.

## Discussion

We report the observation of superballistic transport in a 2DEG, driven by the constructive EMP interference under MW irradiation. Our central finding is that constructive EMP interference induces a sharp enhancement of positive photoconductance, whereas the effects of destructive interference are remarkably suppressed. This phenomenon-pronounced signal from constructive interference alongside the quenching of destructive interference effects—reveals a specific mechanism for controlling electron flow.

The superballistic nature of this transport is unambiguously established through multiple lines of evidence: (1) a photoconductance that scales quadratically with the PC width $W$, (2) the characteristic suppression of superballistic transport by a magnetic field, and (3) the MW power dependence consistent with hydrodynamic heating models. The ${W}^{2}$ scaling of the photoconductance and its suppression by magnetic fields provide definitive signatures of electron–electron collisions, confirming the hydrodynamic origin of the observed phenomena.

We have considered several alternative mechanisms that could potentially influence the observed superballistic transport, including thermoelectric effects^53^ and Hall viscosity^15,54^. Thermoelectric signals, which could arise from the temperature gradient near the PCs, are mostly filtered out by our use of a lock-in measurement technique, which selectively detects the AC resistance (see SI for more details). However, we cannot rule out the possibility that the thermoelectric effect may still have some influence in our experiments, given that thermal gradients compete with viscous effects in the governing Navier–Stokes equations. The potential influence of Hall viscosity can be dismissed. Its contribution is inherently weak in our symmetric PC geometry (see SI for more details). Furthermore, Hall viscosity does not directly contribute to standard linear-response transport coefficients, such as resistance, making its signature difficult to detect in our measurements.

Our work deepens the understanding of EMP physics and significantly advances the study of hydrodynamic electron transport in nanoscale constrictions. These findings open an avenue for exploring collective electron dynamics and viscous flow in mesoscopic systems. While a concurrent independent study^33^ has explored superballistic transport in graphene under terahertz radiation at zero magnetic field, our work uniquely demonstrates these effects in a finite magnetic field. Magneto-transport reveals clear interference phenomena and provides access to a richer physical regime, underscoring the role of electron orbital dynamics in hydrodynamic electron transport.

## Methods

### Experimental setup and sample details

Our experiments were performed in a ^3^He refrigerator with a base temperature of 0.3 K. The samples were fabricated from two wafers: wafer A, hosting a 2DEG with an ultrahigh mobility of $\mu \sim 2\times {10}^{7}\,{{{\rm{cm}}}}^{2}/{{\rm{Vs}}}$, and wafer B, with a high mobility of $\mu \sim 2\times {10}^{6}\,{{{\rm{cm}}}}^{2}/{{\rm{Vs}}}$. Both wafers were grown by molecular beam epitaxy and feature a standard GaAs/AlGaAs quantum well structure commonly used in such systems. Modulation doping was employed to introduce silicon dopants into the GaAs quantum well, enabling the formation of high-mobility 2DEGs. Each wafer had a carrier density of $2.6\times {10}^{11}\,{{{\rm{cm}}}}^{-2}$, measured at 300 mK after brief illumination with a red light-emitting diode.

Two types of PCs were used in this study, both yielding similar experimental results. The first type was a conventional split-gate PC defined by electron-beam lithography and a Ti/Au top gate, while the second type was fabricated by wet etching down to the buffer layer. Electrical contacts were formed using a Ge/Pd/Au alloy annealed at 450 °C in a H_2_/N_2_ atmosphere. Detailed sample parameters are provided in Table 1.

**Table 1:** Details of all the PCs, including PC type, carrier density, mobility at *T* = 0.3 K and geometric width of PCs

	Type of PCs	$n/{{\rm{c}}}{{{\rm{m}}}}^{-2}$	$\mu /{{\rm{c}}}{{{\rm{m}}}}^{2}{{{\rm{V}}}}^{-1}{{{\rm{s}}}}^{-1}$	$W/{{\rm{\mu }}}{{\rm{m}}}$
PC A1	Split-gate	$2.5\times {10}^{11}$	$2.4\times {10}^{7}$	1.2
PC A2	Split-gate	$2.5\times {10}^{11}$	$2.4\times {10}^{7}$	0.92
PC A3	Etched	$2.5\times {10}^{11}$	$2.4\times {10}^{7}$	0.18
PC A4	Etched	$2.5\times {10}^{11}$	$2.4\times {10}^{7}$	1.1
PC A5	Etched	$2.5\times {10}^{11}$	$2.4\times {10}^{7}$	2.0
PC A6	Etched	$2.5\times {10}^{11}$	$2.4\times {10}^{7}$	3.2
PC A7	Split-gate	$2.5\times {10}^{11}$	$2.4\times {10}^{7}$	0.64
PC B1	Split-gate	$2.6\times {10}^{11}$	$2.2\times {10}^{6}$	1.0
PC B2	Split-gate	$2.6\times {10}^{11}$	$2.2\times {10}^{6}$	0.61

The linear conductance was measured using a lock-in technique, which detects the in-phase voltage response to an AC current. This approach effectively filters out DC thermoelectric contributions. MW irradiation was supplied by a Gunn-effect source with frequencies ranging from 30 to 105 GHz, and the MW power was finely adjusted using a rotary vane attenuator. The applied MW irradiation was nondestructive and was cycled on and off during measurements. Its sole purpose was to excite the electron system via EMPs, resulting in electron heating. This process is fully reversible and does not alter the intrinsic disorder of the sample.

Here we provide the structural details of the PCs used in our experiments. Samples PC A1–A7 were fabricated from the ultrahigh-mobility wafer A, while PC B1–B2 were prepared from the high-mobility wafer B. The effective PC width $W$ was determined using the Sharvin formula. For split-gate PCs, whose width is electrically tunable, $W$ is defined as the maximum width achievable just before the electron gas beneath the split gates is fully depleted. All PCs share the same geometric length along the transport direction of the constriction, with ${l}_{{\mbox{PC}}}=400{\mbox{nm}}$.

### Determination of the effective PC size

**Fig. 6:**
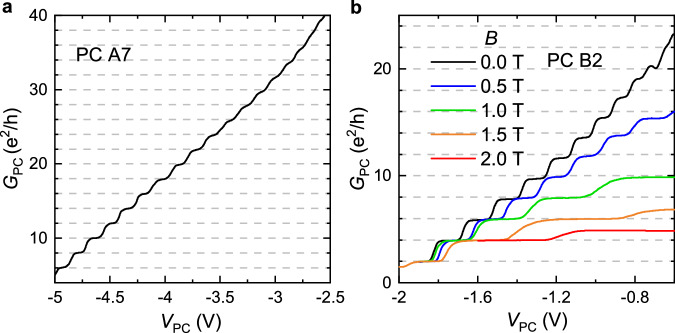
Quantized conductance of quantum point contacts. **a** Conductance ${G}_{{{\rm{PC}}}}$ of quantum point contact PC A7 as a function of split-gate voltage ${V}_{{\mathrm{PC}}}$ at zero magnetic field. The linear dependence of ${G}_{{{\rm{PC}}}}$ on ${V}_{{{\rm{PC}}}}$ during electron depletion beneath the split gates indicates that Sharvin’s formula provides a reliable estimate of the effective width $W$ even for wide PCs. **b** Quantized conduction plateaus are demonstrated in PC B2 under varying magnetic fields, confirming the high quality of the quantum point contact.

The effective electrical width $W$ is determined by Sharvin’s formula^25^: $W={{{\rm{\pi }}}{{\rm{h}}}/2{{{\rm{e}}}}^{2}\sqrt{2{{\rm{\pi }}}n}R}_{{{\rm{L}}},0}(B=0)$. Split-gate PCs exhibit quantized conductance when only a few conductance channels are present, confirming their behavior as quantum PCs in the narrow regime (Fig. 6a, b)^26^. The high quality of these PCs is evidenced by well-defined quantized plateaus, as shown in Fig. 6b. Even for slightly wider PCs, Sharvin’s formula provides a reliable estimate of $W$. For example, in PC A7 (Fig. 1b), an emerging conductance plateau near ${N}_{{\mbox{PC}}}=18$ corresponds to *G*_PC _≈ 35.6e^2̸^h, indicating an effective width of 0.44 μm with an error below 1.5%. This small deviation is attributed to the ultrahigh mobility of our samples, which ensures that the PC resistance is dominated by the number of conductance channels ${N}_{{\mbox{PC}}}$.

Given that ${G}_{{\mbox{PC}}}$ varies linearly with gate voltage ${V}_{{\mbox{PC}}}$ until full depletion under the split gates, we conclude that Sharvin’s formula yields accurate estimates of $W$ when the effective PC size is smaller than its geometric dimensions. Our analysis confirms that the formula remains reliable for W < 1.5  μm^55^. The calculated effective widths for the PCs listed in Table1 are *W *= 1.17, 0.92, 0.64, 1.0, 0.61 μm for PCs A1, A2, A7, B1, and B2, respectively. These values closely match the corresponding geometric split-gate spacings *W*_*g*_ = 1.2, 1.0, 0.6, 1.0, and 0.6 μm, further validating that the effective width derived from PC resistance is sufficiently accurate for hydrodynamic size-dependence analysis.

### Distinguishing local heating from global thermalization

**Fig. 7:**
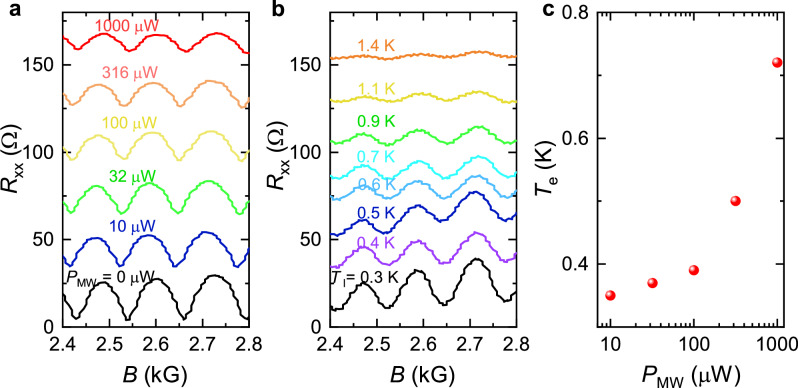
Electron temperature in the two-dimensional electron gas. **a** SdH oscillations in the 2DEG of wafer A under MW irradiation. **b** SdH oscillations measured in the same sample (PC A6) at different bath temperatures. **c** Comparison between panels a and b enables extraction of the electron temperature ${T}_{{{\rm{e}}}}$ in the 2DEG, which is significantly lower than that in the PC region.

MW radiation can heat the electrons in the narrow constrictions to ${T}_{{{\rm{e}}}}\gg 1{{\rm{K}}}$, driving them into the hydrodynamic regime, while the bulk 2DEG remains cold ($< 1\,{{\rm{K}}}$). The most direct evidence comes from monitoring the SdH oscillations in the bulk resistance. Under strong MW irradiation (1000 μW), we still observe pronounced SdH oscillations at low magnetic fields of 2.4–2.8 kG (Fig. 7a). In contrast, these oscillations are known to be suppressed at temperatures above 1 K (Fig. 7b). The persistence of SdH oscillations under irradiation indicates that the global electron and lattice temperatures, ${T}_{{{\rm{l}}}}$, remain well below 1 K. Although some local dissipation is expected, these results confirm that the applied MW power does not cause substantial global heating of the 2DEG. Figure 7c shows the electron temperature in the 2DEG under MW irradiation, determined using the method described earlier.

To provide a qualitative comparison with data obtained under MW irradiation, we measured the longitudinal resistance ${R}_{{\mbox{L}}}$ at various bath temperatures (e.g., Fig. S5 in SI). While the overall trends are similar, it is important to note that bath temperature variations cannot be used to directly calibrate the electron temperature under MW irradiation. The key difference lies in the heating profiles: under MW exposure, Joule heating is highly localized, raising the temperature in the PC region to approximately 10 K while the bulk 2DEG remains below 1 K. In contrast, increasing the bath temperature heats both the 2DEG and the PC region uniformly.

This fundamental difference leads to significant disparities that complicate the accurate determination of the electron temperature ${T}_{{{\rm{e}}}}$ under MW irradiation. Specifically, the measured viscous conductance ${G}_{{{\rm{v}}}}$ is difficult to extract due to the influence of the bulk 2DEG resistance, which depends on the flow geometry near the PCs. The total PC resistance is given by ${R}_{{\mbox{PC}}}={({G}_{0}+{G}_{{{\rm{v}}}})}^{-1}+b{\rho }_{{{\rm{xx}}}},$ where $b$ is a numerical coefficient that quantifies the contribution of the bulk 2DEG near the PC region, and ${\rho }_{{{\rm{xx}}}}$ is the 2DEG resistivity. The primary difficulty arises from the uncertainty in $b$ at finite magnetic fields. This is why the bath-temperature calibration (e.g., Fig. S5) cannot be directly used to determine ${T}_{{{\rm{e}}}}$ in the PC region under MW irradiation.

### Simulation of electron temperature ${{{\boldsymbol{T}}}}_{{{\bf{e}}}}$ and longitudinal resistance ${{{\boldsymbol{R}}}}_{{{\bf{L}}}}$ based on the edge magnetoplasmons and hydrodynamic model

We present simulation results for the electron temperature ${T}_{{{\rm{e}}}}$ and longitudinal resistance ${R}_{{\mbox{L}}}$ based on both the EMP model and the hydrodynamic model. The EMP model accurately accounts for the observed resistance oscillations, while the hydrodynamic model explains the superballistic transport behavior and the influence of ${T}_{{{\rm{e}}}}$ on ${R}_{{\mbox{L}}}$.

In the EMP model, the wave vector $q$ is calculated at each magnetic field according to the EMP dispersion relation. We account for interference between two sets of oscillations. The absorbed MW power is given by ${P}_{{{\rm{a}}}}\propto |{1+\exp ({{\rm{i}}}q\Delta {L}_{1})+\exp ({{\rm{i}}}q\Delta {L}_{2})|}^{2},$ where $\Delta {L}_{1}$ and $\Delta {L}_{2}$ represent the propagation length differences for the two oscillation sets. Using the empirical relation ${T}_{{{\rm{e}}}}\propto {P}_{{{\rm{a}}}}^{0.56}$ obtained from our experiments, we express the electron temperature as ${T}_{{{\rm{e}}}}={T}_{0}|{1+ \exp ({{\rm{i}}}q\Delta {L}_{1})+\exp ({{\rm{i}}}q\Delta {L}_{2})|}^{1.12},$ where ${T}_{0}$ depends on the actual MW power emitted by the source.

The EMP model successfully reproduces the relative strength of ${P}_{{{\rm{a}}}}$ under constructive interference conditions. As shown in Fig. 5c, the amplitude of the temperature peak can be finely adjusted by varying $\Delta {L}_{1}$. Despite the pronounced contrast between the two traces, the only differing parameter is $\Delta {L}_{1}$, which is set to 500 and 550 μm, respectively, while $\Delta {L}_{2}$ is fixed at 1150 μm. The value of $\Delta {L}_{2}$ is chosen to match the oscillation period of the measured ${R}_{{\mbox{L}}}$, whereas $\Delta {L}_{1}$ primarily affects the abrupt changes in resistance. This sensitivity to small variations in $\Delta {L}_{1}$ or $\Delta {L}_{2}$ explains the marked asymmetry of the traces with respect to the magnetic field.

For the hydrodynamic model, we compute ${R}_{{\mbox{L}}}$ from ${T}_{{{\rm{e}}}}$ using Eqs. 1 and 2, after selecting an appropriate ${T}_{0}$. A minor correction to Eq. 2 is necessary near zero magnetic field, where the original expression becomes inadequate. To avoid divergence in the viscous scattering time ${\tau }_{{{\rm{ee}}}}$ when the condition ${{{\rm{k}}}}_{{{\rm{B}}}}^{2}{T}_{{{\rm{e}}}}^{2}/{E}_{{{\rm{F}}}}^{2}\ll \beta$ is violated, we apply a cutoff at $B=0.2{\mbox{kG}}$.

The simulation parameters are as follows: MW frequency $f=72{\mbox{GHz}}$, electron–electroninteraction strength ${\alpha }_{{{\rm{ee}}}}=0.5$, PC width $W=540{\mbox{nm}}$, and ${T}_{0}=3.0{\mbox{K}}$ for an input power of ${P}_{{{\rm{MW}}}}=316\,{{\rm{\mu }}}{{\rm{W}}}$. The value of ${T}_{0}$ under different irradiation levels can be scaled according to ${T}_{0}\propto {P}_{{\mbox{MW}}}^{0.56}$.

### Estimation of key parameters in hydrodynamic models

We estimate several key parameters to compare with hydrodynamic theoretical predictions. As an example, the calculated parameters for samples PC A1–A3 are listed in Table 2.

**Table 2:** PC width ${{\boldsymbol{W}}}$, electron–electron scattering length ${{{\boldsymbol{l}}}}_{{{\bf{ee}}}}$ and viscous flow length scale ${{{\boldsymbol{D}}}}_{{{\bf{v}}}}$ (at ${{{\boldsymbol{T}}}}_{{{\bf{e}}}}\sim {{\boldsymbol{10}}}\,{{\bf{K}}}$) of PC A1.a, A1.b, A2.a, A2.b, A3

	$W/{{\rm{\mu }}}{{\rm{m}}}$	${l}_{{ee}}/{{\rm{\mu }}}{{\rm{m}}}$	${D}_{v}/{{\rm{\mu }}}{{\rm{m}}}$
PC A1.a	1.2	«1.6	«3.0
PC A1.b	0.92	«1.2	«2.6
PC A2.a	0.82	«1.5	«2.9
PC A2.b	0.54	«0.84	«2.2
PC A3	0.18	«0.77	«2.1

The first parameter is the viscous scattering length, defined as ${l}_{{{\rm{ee}}}}=\sqrt{2{{\rm{\pi }}}n}{{{\rm{e}}}}^{2}{W}^{2}/16\hslash {G}_{{{\rm{v}}}}(B=0),$ which is evaluated when hydrodynamic effects dominate (i.e., at ${T}_{{{\rm{e}}}}\sim 10{\mbox{K}}$)^6,8,10–12^. Based on the experimental data in Fig. 3, we compute ${l}_{{{\rm{ee}}}}$ at $B=0.5{\mbox{kG}}$ near the positive-to-negative transition of the photoconductance (Table 2). Since hydrodynamic behavior is suppressed under finite magnetic fields, the estimated ${l}_{{{\rm{ee}}}}$ at $B=0.5{\mbox{kG}}$ is significantly larger than its zero-field value. Nevertheless, the zero-field viscous scattering length is comparable to or even smaller than the PC width $W$, indicating that at ${T}_{{{\rm{e}}}}=10{\mbox{K}}$ the PCs operate in or near the hydrodynamic regime. At lower temperatures or under weaker MW irradiation, ${l}_{{{\rm{ee}}}}$ remains close to this regime. Superballistic transport does not require an extreme hydrodynamic limit (${l}_{{{\rm{ee}}}}\ll W$), and viscous effects can still manifest near the ballistic regime (${l}_{{{\rm{ee}}}}\gg W$), consistent with our experimental observations.

The second parameter, the viscous flow length scale ${D}_{{{\rm{v}}}}=\sqrt{{l}_{{{\rm{ee}}}}{l}_{0}}/2,$ is also overestimated at finite magnetic fields^52^. The values in Table2 are derived from data at $B=0.5{\mbox{kG}}$. At $B=1{\mbox{kG}}$, the cyclotron radius ${R}_{{{\rm{c}}}}={m}^{*}{v}_{{{\rm{F}}}}/{{\rm{e}}}B=0.82\,{{\rm{\mu }}}{{\rm{m}}}$ is close to ${D}_{{{\rm{v}}}}$, suggesting that at low magnetic fields the PCs operate in or near the hydrodynamic regime. Our observation of positive photoconductance is thus consistent with hydrodynamic theory.

## Supplementary information

Supplementary Information

Transparent Peer Review File

## Data Availability

All the data generated in this study have been deposited in the *figshare* database under accession code 10.6084/m9.figshare.29653481.
