# Supplementary material for: Superballistic flow of viscous electron fluid induced by edge magnetoplasmons in point contacts
Source: Nat Commun. 2026 Jun 6;17:7238. doi: 10.1038/s41467-026-73154-5 (PMC13396176; doi:10.1038/s41467-026-73154-5)
Supplement: Supplementary file 1 — Supplementary Information [file 41467_2026_73154_MOESM1_ESM.pdf]

Supplemental material to 'Superballistic flow of viscous electron fluid induced by edge magnetoplasmons in point contacts'

Xinghao Wang[*], Wenfeng Zhang and Rui-Rui Du[**]

International Center for Quantum Materials, School of Physics, Peking University, Beijing 100871, China

L. N. Pfeiffer, K. W. Baldwin, and K. W. West

Department of Electrical Engineering, Princeton University, Princeton, NJ 08544, USA

I. Signatures of edge magnetoplasmons

In this section, we present multiple lines of evidence demonstrating that the magnetoresistance oscillations observed in our point contacts (PCs) originate from edge magnetoplasmons (EMPs). As shown in Fig. S1b, the resistance oscillates periodically with magnetic field B. Moreover, Fig. S1a reveals that the magnetic field period of these EMP-induced resistance oscillations (EIROs) scales inversely with the microwave (MW) frequency f, a characteristic signature of EMPs.

The observed oscillations are MW-induced, which raises the possibility of origins such as $1/B$-periodic MW-induced resistance oscillations (MIROs) [1-4] or bulk/edge magnetoplasmons. However, the B-periodic nature of the oscillations rules out MIROs. Furthermore, in split-gate samples, when electrons are not depleted beneath the gates, the B-periodic oscillations vanish and MIRO signals appear instead (Fig. S1b). These observations collectively indicate that EMPs are the sole plausible mechanism responsible for the observed resistance oscillations.

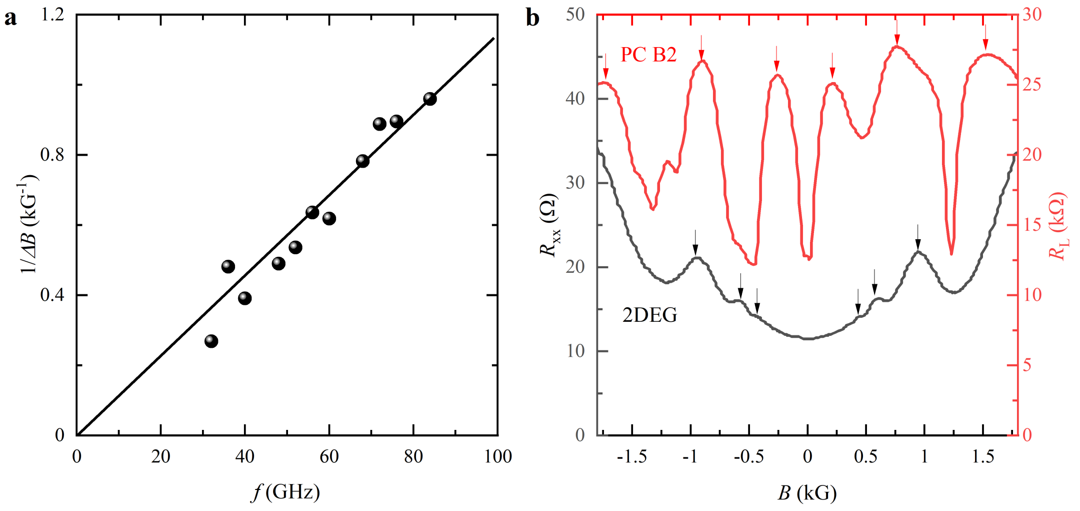

Fig. S1 | **Periodicity of EIROs and its comparison with MIROs. a** Period of resistance oscillation (the large period one) for traces with different MW frequency is measured and $1/\Delta B$ is proportional to f. This is direct evidence of EMPs. The solid line represents the best-fit line constrained to pass through the origin. **b** Under MW radiation of $f = 71$ GHz and $P_{\mathrm{MW}} = 10000$ μW, PC B2 shows totally different traces when split-gate voltage is applied (red line) or not (black line). Arrows marking the maxima shows B- and $1/B$-periodic resistance oscillation for EIRO and MIRO.

For PCs in the open regime (PC conductance $G_{\mathrm{PC}} > \mathrm{e}^2/\mathrm{h}$), previous experiments have shown that conductance decreases under MW irradiation [5]. This behavior is also observed in our measurements under sufficiently high MW power, while the unusual features at weak irradiation are attributed to hydrodynamic effects. Here, we focus on the high-power regime and disregard contributions from superballistic transport.

As shown in Fig. 2c of the main text, the resistance peaks grow in amplitude with increasing MW power and eventually saturate near 32 kΩ. Notably, regardless of irradiation intensity, certain "fixed points" retain their original resistance values, indicating perfect destructive interference at specific magnetic fields.

EIROs exhibit strong robustness against thermal excitation due to their origin in magnetoplasmons, which persist up to several tens of kelvin. As seen in Fig. S2a,

oscillations remain pronounced even near 20 K. Figure S2b illustrates the evolution of EIROs with PC size: at $V_{PC} = -1.52$ V, PC B2 approaches the crossover between the open and tunneling regimes, while at $V_{PC} = -0.90$ V, the split gate has just depleted electrons beneath it, resulting in EIROs that are more sensitive to MW radiation. In both cases, prominent resistance peaks persist as the split-gate voltage varies.

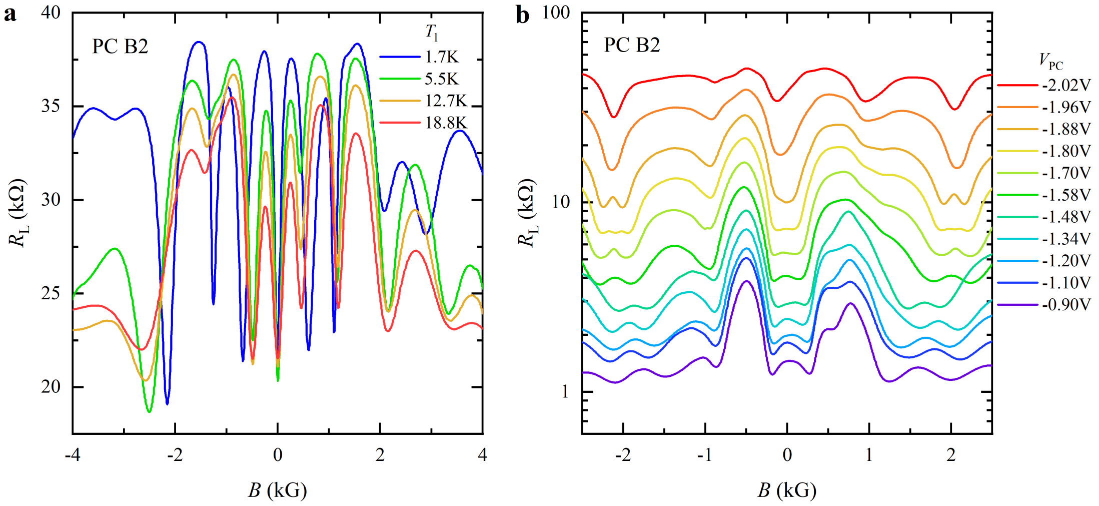

Fig. S2 | **Temperature and split-gate-voltage dependence of EIROs. a** Temperature-dependence of EIROs indicates that EMPs are robust against heating (PC B2, $f = 71$ GHz and $P_{MW} = 10000$ μW). **b** EIROs evolves with PC width, continuously tuned via split-gate voltage V_{PC} (PC B2, $f = 57$ GHz and $P_{MW} = 10000$ μW).

II. Thermoelectric effect

Apart from the hydrodynamic interpretation, an alternative explanation for the observed positive photoconductance in point contacts could involve thermoelectric effects. When a PC is locally heated by interfering EMPs, a significant temperature gradient may form across the constriction, potentially generating a thermoelectric voltage along the PC direction. This signal would also be expected to depend on the magnetic field, given its origin in EMP excitation.

However, due to the symmetric geometry of our PC devices, any thermoelectric voltages generated at the two ends of the constriction should largely cancel out. Residual signals could still arise from fabrication imperfections or sample inhomogeneities. If the observed positive photoconductance were dominated by such residual thermoelectric contributions, its sign would reverse when the current direction is inverted - since the thermoelectric voltage does not change polarity with current flow. This is not observed in our experiments.

Additionally, we directly measured potential thermoelectric signals using lock-in techniques and found the voltage difference across the PC under MW irradiation to be negligible. The lock-in method inherently filters out DC thermoelectric components, further confirming that thermoelectric effects do not play a significant role in the photoconductance behavior reported here.

III. From PCs to normal Hall bars

As the PC width increases, the influence of EMPs on magnetoresistance oscillations progressively diminishes. Under fixed MW power, EIROs gradually fade as the PC geometry transitions toward that of a conventional Hall bar.

Fig. S3 illustrates typical $1/B$-periodic MIRO signals measured in a $W = 3.2$ μm PC (PC A6), which essentially functions as a narrow Hall bar at the center of the device. The measured traces under MW irradiation clearly exhibit MIRO characteristics, accompanied by a zero-field resistance peak. The latter is a hallmark of PC or narrow Hall bar geometries, while the former originates from bulk 2DEG transport.

It should be noted that magnetoresistance measurements become less accurate when the PC resistance is small, as the contribution from the surrounding 2DEG region becomes non-negligible and affects the extracted PC resistance values.

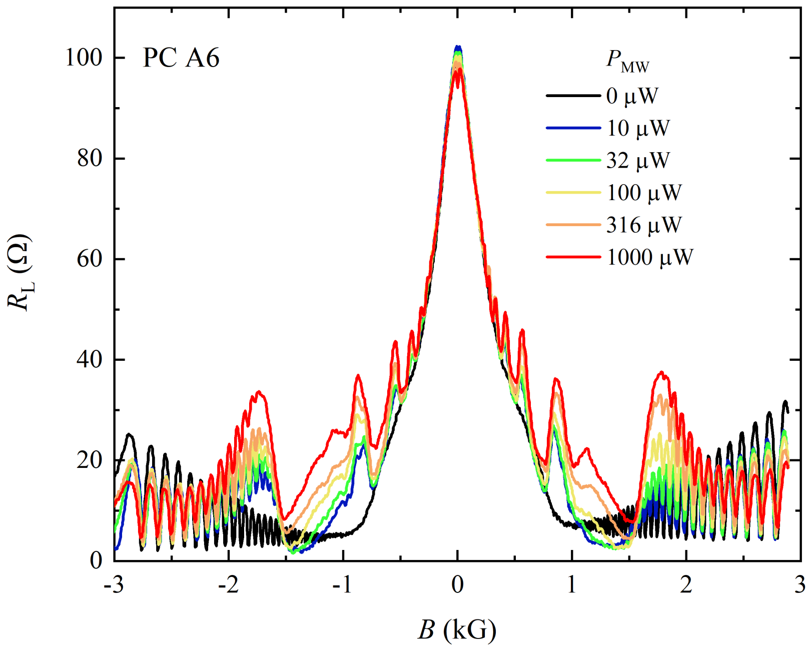

Fig. S3 | **Wide PCs do not show EIROs.** PC A6 is the widest PC in our experiments with $W =$ 3.2 μm. Its trace in the dark (black line) showing negative magnetoresistance is similar to those of narrow Hall bars. Due to its low resistance, this PC is not as sensitive to MW radiation as narrow PCs so that no EIRO is observed at $f = 71$ GHz and $P_{MW} = 10000$ μW. Instead, small signal of MIROs originating from 2DEG beside the PC dominates in this sample.

IV. Negative photoconductance

In the phonon-scattering regime, where electron–phonon interactions dominate transport, we can estimate the electron temperature T_e from the large resistance peaks observed. The PC resistance R_{PC}, measured via the diagonal resistance R_D, is modeled from ballistic to diffusive transport regimes [6]. It is well established that $R_{PC} = R_b + R_d$, with the ballistic and Drude contributions given by

$$R_b = h/2e^2 N_{PC}\,, \tag{1}$$

$$R_d = \frac{h}{2e^2}\frac{2}{\sqrt{2\pi n}\,l_0}\frac{l_{PC}}{W}\,, \tag{2}$$

where l_0 is the impurity scattering mean free path, N_{PC} is the number of ballistic channels, and $l_{PC} = 400$ nm is the PC propagation length.

Given the complex scattering mechanisms in GaAs quantum wells - including acoustic and optical phonon scattering - we apply an approximate exponential relation between T_e and l_0, following Refs. [7,8]. Using the measured longitudinal resistance R_L, we extract l_0 and thereby estimate T_e in the phonon-scattering regime at the resistance peaks. Under strong MW irradiation (e.g., $P_{MW} \sim 10000$ μW), the mean free path satisfies $l_0 \ll l_{PC}$, and T_e can exceed 100 K, as illustrated in Fig. S4. This explains the robustness of EIROs at elevated temperatures.

In addition to the above interpretation of negative photoconductance, we note that EMPs themselves may also contribute to negative photoconductance in PCs operating in the open regime. However, distinguishing whether resistance peaks are dominated by phonon scattering or EMP-induced voltages falls beyond the scope of this work.

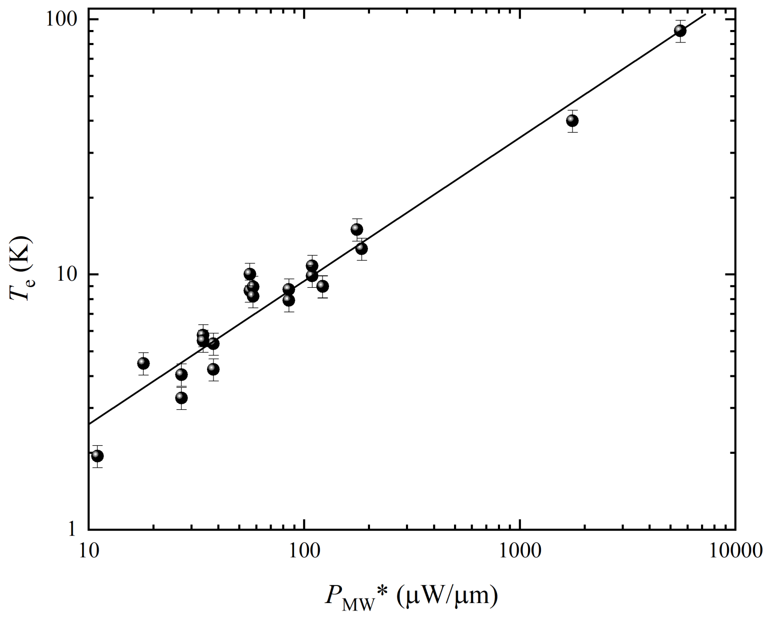

Fig. S4 | T_e versus P^*_{MW} including two points in the phonon-scattering regime. Two estimated T_e points in phonon-scattering regime from resistance peaks of EIROs are shown. They also fit with

$T_e \propto {P^*_{MW}}^{0.56}$ quite well. The dominant uncertainty in T_e arises from the uncertainty in the electron-electron interaction parameter α_{ee}, shown as the error bars representing the standard error (SE). The solid line represents the best-fit line.

In Fig. 4b of the main text, the estimated electron temperature T_e saturates at approximately 15 K for PCs of various widths - corresponding to the temperature at which EIRO peaks start to appear. This saturation behavior can be explained as follows.

The transition from positive to negative photoconductance at constructive interference magnetic fields is expected to occur at similar T_e values across different PCs, since this crossover marks the shift from superballistic to phonon-scattering dominated transport. As indicated in Ref. [9], the electron temperature at this transition is largely insensitive to geometric parameters such as PC width.

As resistance peaks evolve from dips in the measured traces, the hydrodynamic model used in our main text - which does not incorporate phonon scattering - overestimates T_e and leads to an apparent saturation near 10-15 K. In reality, this saturation is an artifact of the model's limitations rather than a true physical saturation of electron temperature. A more accurate estimation of T_e would require a comprehensive model that simultaneously accounts for both hydrodynamic transport and electron–phonon scattering, though such a treatment would be considerably more complex.

V. Hall viscosity

Under a static magnetic field, the viscous response of a charged fluid is governed by a tensorial term in the Navier–Stokes equation, which includes a dissipationless off-diagonal component known as the Hall viscosity [10,11]. The Hall viscosity contributes a magnetic-field-linear correction to the resistance, opposite in sign to the classical Hall

effect. However, its signatures are not prominent in our experimental data for several system-specific reasons.

Hall viscosity is challenging to probe directly, as it does not enter standard linear-response transport coefficients such as resistance. Experimental detection typically relies on secondary effects - for example, boundary corrections to the resistance near current injection points [12]. In the context of a PC, the Hall viscosity manifests at both ends of the constriction. However, in a symmetric PC geometry, the electrical signals generated at the upstream and downstream edges are opposite in polarity. As a result, they tend to cancel in a two-terminal voltage measurement, leading to a negligible net signal.

This interpretation is consistent with the measured longitudinal resistance R_L traces, which show only minimal asymmetry with respect to the magnetic field (Fig. S5). In principle, weak Hall viscosity signals could become detectable in geometrically asymmetric PCs. Furthermore, in our high-mobility GaAs/AlGaAs heterostructure under magnetic fields on the order of 0.5-1 kG, the ratio of Hall viscosity to shear viscosity is estimated to be relatively small. Consequently, its contribution to the total conductance is likely obscured by the dominant effects of conventional viscosity.

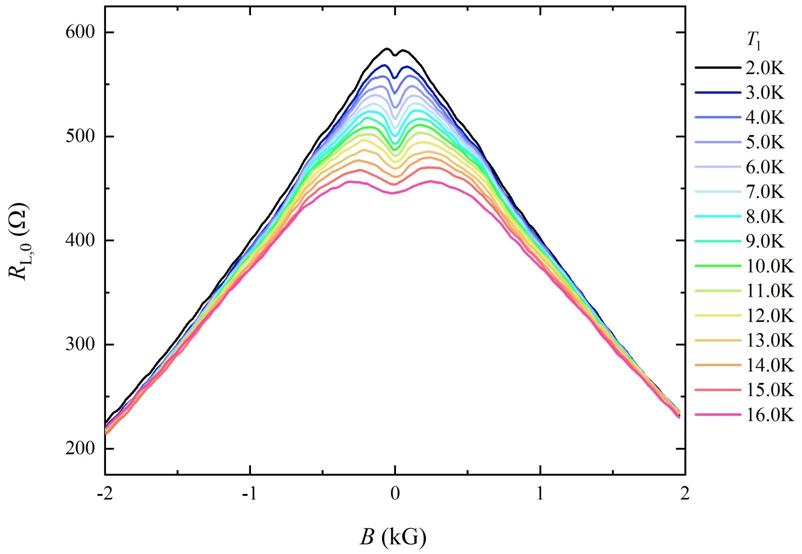

Fig. S5 | Temperature dependence of $R_{\mathrm{L,0}}$ subtracted with contribution from 2DEG resistance. No evident signal of Hall viscosity is observed in our PCs, e.g., PC A2 b, since $R_{\mathrm{L,0}}$ is symmetric with B. The reduced resistance due to viscosity conductance is comparable to the data from the measurments under MW irradiation.

VI. Determine the dispersion relation of edge magnetoplasmons

According to Ref. [13,14], the spectrum of EMPs propagating along the boundary of a semi-infinite 2DEG is governed by the equation

$$\frac{\mathrm{i}|q|\sigma_{\mathrm{xx}}(\omega)}{q\sigma_{\mathrm{yx}}(\omega)} - \tanh\left(\int_0^{\frac{\pi}{2}} \ln\left(\epsilon\left(\frac{|q|}{\sin t}, \omega\right)\right) \frac{dt}{\pi}\right) = 0, \tag{3}$$

where $\sigma_{\alpha\beta}(\omega)$ is the AC Drude conductivity tensor of the 2DEG, and the effective dielectric function

$$\epsilon(q,\omega) = 1 + \frac{4\pi\mathrm{i}\sigma_{\mathrm{xx}}(\omega)q}{\omega\epsilon_{\mathrm{r}}\left(\frac{\epsilon_{\mathrm{r}}\tanh(qd)+1}{\epsilon_{\mathrm{r}}+\tanh(qd)}+1\right)}, \tag{4}$$

depends on the dielectric constant ϵ_{r} and the thickness $d = 200$ nm from the 2DEG layer to the sample surface. The numerical solution to the Eqs. (3) - (4) yields the EMP dispersion relation, which shows that the wave vector q scales such that q/ω varies linearly with both ω and ω_{c}. At zero magnetic field, the 2D plasmon dispersion relation follows $q \propto \omega^2$.

VII. Heat flow through point contacts

For narrow constrictions such as PCs, the thermal conductance K in the near-ballistic transport regime scales linearly with the electron temperature T_{e}. Specifically, in the ballistic limit, $K = N_{\mathrm{PC}} \cdot \pi k_{\mathrm{B}}^2 T_{\mathrm{e}}/3\hbar$ [15], consistent with the Wiedemann-Franz law. In our experiment, the PCs were heated by EMPs, establishing a temperature gradient between the PC region and the bulk 2DEG. Consequently, the heat flow between these regions can be expressed as $P_{\mathrm{tr}} = K\nabla T_{\mathrm{e}} \approx \pi k_{\mathrm{B}}^2 N_{\mathrm{PC}}(T_{\mathrm{e}}^2 - T_{\mathrm{b}}^2)/6\hbar l_{\mathrm{PC}}$ (the Peltier term cancels out). Here, the bulk 2DEG rather than the lattice acts as the dominated cold bath for hot electrons in the PC, because electron-phonon coupling is relatively weak at lattice temperatures around 0.3 K. This explains the observed quadratic dependence $P_{\mathrm{a}} \propto T_{\mathrm{e}}^2$, leading to $T_{\mathrm{e}} \propto P_{\mathrm{a}}^{0.5}$. These results indicate the presence of a dominant electron-mediated heat relaxation channel for hot electrons within the PCs.

References

[1] Tkachenko, V. A. et al. Low-frequency microwave response of a quantum point contact. *JETP Letters* **114**, 2, 110–115 (2021).

[2] de Jong, M. J. M. Transition from Sharvin to Drude resistance in high-mobility wires. *Phys. Rev. B* **49,** 7778–7781 (1994).

[3] Pheiffer, L. et al. The role of MBE in recent quantum Hall effect physics discoveries. *Physica E* **20**, 57–64 (2003).

[4] Harris, J. J. et al. Acoustic phonon scattering in ultrahigh mobility, low carrier density GaAs/(Al,Ga)As heterojunctions. *Surf. Sci.* **229**, 113-115 (1990).

[5] Kumar, R. K. et al. Superballistic flow of viscous electron fluid through graphene constrictions. *Nat. Phys.* **13**, 1182 (2017).

[6] Avron, J. E. Odd viscosity. *J. Stat. Phys.* **92**, 543–557 (1998).

[7] Sherafati, M., Principi, A., and Vignale, G. Hall viscosity and electromagnetic response of electrons in graphene. *Phys. Rev. B* **94**, 125427 (2016).

[8] Berdyugin, A. I. et al. Measuring Hall viscosity of graphene's electron fluid. *Science* **364**, 162-165 (2019).

[9] Zudov, M. A. et al. Shubnikov–de Haas-like oscillations in millimeterwave photoconductivity in a high-mobility two-dimensional electron gas. *Phys. Rev. B* **64**, 201311(R) (2001).

[10] Ye, P. D. et al. Giant microwave photoresistance of two-dimensional electron gas. *Appl. Phys. Lett.* **79**, 2193 (2001).

[11] Mani, R. G. et al. Zero-resistance states induced by electromagnetic-wave excitation in GaAs/AlGaAs heterostructures. *Nature* **420**, 646 (2002).

[12] Zudov, M. A. et al. Evidence for a new dissipationless effect in 2D electronic transport. *Phys. Rev. Lett.* **90**, 046807 (2003).

[13] Volkov, V. A. & Mikhailov, S. A. Edge magnetoplasmons: low frequency weakly damped excitations in inhomogeneous two-dimensional electron systems. *Sov. Phys. JETP* **67**, 1639 (1988).

[14] Levin, A. D. et. al. Giant microwave-induced *B*-periodic magnetoresistance oscillations in a two-dimensional electron gas with a bridged-gate tunnel point contact. *Phys. Rev. B* **95**, 081408(R) (2017).

[15] Proetto, C. R., Heat conduction through ballistic quantum-point contacts: Quantized steps in the thermal conductance. *Solid State Commun.* **80**(11): 909-912 (1991).
