# Supplementary material for: Superballistic flow of viscous electron fluid induced by edge magnetoplasmons in point contacts
Source: Nat Commun. 2026 Jun 6;17:7238. doi: 10.1038/s41467-026-73154-5 (PMC13396176; doi:10.1038/s41467-026-73154-5)
Supplement: Supplementary file 2 — Transparent Peer Review File [file 41467_2026_73154_MOESM2_ESM.pdf]

nature portfolio

Peer Review File

Superballistic flow of viscous electron fluid induced by edge magnetoplasmons in point contacts

Corresponding Author: Mr Xinghao Wang

This file contains all reviewer reports in order by version, followed by all author rebuttals in order by version.

Version 0:

Reviewer comments:

Reviewer #1

(Remarks to the Author)
The manuscript reports experiments on superballistic flow in high-mobility GaAs/AlGaAs point contacts under microwave irradiation, interpreted as arising from constructive interference of edge magnetoplasmons (EMPs) that locally heat electrons and drive them into the hydrodynamic regime. The topic is timely, and the underlying physics is interesting: the connection between EMP interference and viscous transport is actually exciting. However, in its current form the paper is very difficult to follow. The presentation is clouded by filler language, unnecessary detours into tangential topics, and figures that do not clearly emphasize the central message. I do recommend the publication but revision is required to make the work accessible to the broader readership of Nature Communications. Furthermore, the authors need to address the following questions.

Technical questions:

1) Why not show point contact conductance vs regular temperature (by raising the bath T), or at least vs DC bias (to heat electrons)? This would establish the expected non-monotonic dependence (ballistic → superballistic → phonon) without relying only on microwaves first. This would a proof that hydro is there in the first place without EMP. Then the MW power dependence could be compared to this benchmark.

2) The authors assume that microwaves heat only the electron fluid in the constriction, but all absorbed energy must ultimately dissipate to contacts or phonons. How do they ensure that lattice temperature does not rise? What is the cooling power of the cryostat, and how do they know the lattice is thermalized to the bath? Can they provide evidence (e.g. monitoring bulk resistance or thermometer data) that the MW power does not heat the sample globally?

3) The use of logarithmic dBm scale is standard in the microwave community but not intuitive for a general journal audience. Please provide plots in linear power units (mW) in addition to dBm, or at least a conversion.

4) The authors state that the observed scaling of electron temperature with microwave power (T_e~P^0.6) is a signature of phonon cooling. Can the authors justify why they associate the measured exponent of 0.6 with phonon cooling?

5) Magnetic field affects the hydrodynamic flow via Hall viscosity [Science 364, 162-165, 2019] which can also affect the electromagnetic response of 2D fluids [Phys. Rev. B 94, 125427]. Can the authors comment whether this can affect their data or whether any signature of Hall viscosity can be found there.

Other comments:
1) The manuscript suffers from frequent use of vague phrases such as “natural and intriguing effects. These add little substance and obscure the actual results.

2) Figures are dense and not always intuitive. For example, Fig. 2d does contain the superballistic signature, but this is only visible at the lowest MW powers. The key message (ballistic → superballistic → phonon scattering) could be emphasized far more clearly with schematic cartoons or by replotting data to highlight dips vs baseline. The paper is also hard to follow because the same physics is discussed repeatedly in slightly different ways. A tighter structure is needed.

3) The introduction and background cite the authors' previous works intensively, including several MIRO studies that are not directly related to hydrodynamics. MIRO physics is a single-particle, impurity-scattering phenomenon and does not establish viscous flow. At best, MIRO shows that MWs couple to electrons in GaAs. In this paper, the relevant physics is instead EMP-driven heating of constrictions. The narrative should avoid conflating MIRO with hydrodynamics, as this distracts from the real novelty.

Reviewer #2

(Remarks to the Author)
The authors do a set of interesting experiments to look for superballistic conductance in irradiated 2DEGs. The irradiation is supposed to enhance the momentum-conserving scattering rate in a way that was not clear to me but the authors have cited away to other papers. Assuming that this method indeed works, then what the authors propose is potentially intriguing.

However I found the paper to be not well-written, especially to non-technical experts in the field. Some of the comments below are just basic questions that should be more carefully explained in the main text, while others are more basic questions I have about the authors' setup and the reliability of the results. I think these issues need to be addressed; at present I could not recommend the paper for publication.

1) Previous authors have used irradiation to try and add residual resistivity to a sample, i.e. add impurities. It seems however like this is not what the authors are doing here, but they do not really explain what the nature of the irradiation is. Are they trying to damage the sample or is the radiation turned on and off to try and stimulate an additional scattering mechanism on a fixed sample (i.e. that should not be damaged by the radiation).

2) The explanation based on Kohn's Theorem is unclear. Breaking translational symmetry would also presumably allow for momentum-non-conserving collisions. Kohn's Theorem can also be violated by breaking Galilean invariance of a parabolic dispersion relation in a 2DEG, e.g. or by considering the exchange of momentum between phonons and electrons (which together do not have Galilean invariance).

3) I didn't find Equation #1 to be very illustrative (it's also labeled somewhat awkwardly). Is it actually true that the dispersion relation depends on q rather than |q|?

4) To me it is confusing to see a negative P_MW, I think the authors are really reporting \log P_MW and reporting the answer in decibels.

5) What is the value of G_PC in the data shown in Figure 2d? How many multiples of the fundamental unit of conductance is it? This seems to have a very interesting interpretation from the perspective of hydrodynamics, i.e. you might think that N_PC must be large compared to 1 for viscous fluid flow to be relevant (since hydrodynamics depends on multi-particle scattering).

6) The formula #4 is explained in an awkward way. If the magnetic field is extremely small then the set of inequalities above the equation would not hold, since \beta would be extremely small. So actually this formula seems to apply in fairly large magnetic fields.

7) Why can thermoelectric effects be ignored? If the electron system is drastically heating up then this would change the character of hydrodynamic flow. I didn't understand how the lock-in technique filters out these signals. I would think that thermoelectric transport enters the theoretical modeling of the experiment by introducing a new set of boundary conditions on temperature and/or heat current at the contacts. Depending on what these boundary conditions are can have big consequences on the resulting conductivity in hydrodynamics, as was emphasized in the literature on the Wiedemann-Franz law that the authors cite.

8) I was not sure about how the authors estimate the electron temperature, which they say can be very large from the irradiation. The fitting of temperature seems to me to come from the hydrodynamic model itself, but is that not the model the authors are trying to confirm with their analysis? While I could still study the W^2 dependence of conductance it seems that this fitting approach may limit the extent to which the authors can verify another aspect of the hydrodynamic model? Also, the 0.6 exponent relating T and P seems ad hoc to me and not likely to be fundamental.

9) I had a basic physics question about the authors' setup as well. What exactly is the role of the EMP doing here, is it providing additional degrees of freedom to the scatter the electrons off that are flowing far away from the edge? In a narrow PC, how do I cleanly disentangle transport from EMP vs. transport from the hydrodynamic electrons?

Reviewer #3

(Remarks to the Author)
The authors present an experiment on point contacts of two-dimensional electron gases under microwave illumination and magnetic field. They study the microwave photoconductivity of point contacts as a function of magnetic field and observe oscillatory structures. The results are explained in terms of selective heating via edge magnetoplasmons, which push the system in and out of the hydrodynamic regime through enhanced electron–electron collisions.

I find the manuscript to be technically sound, and the results appear reliable. The topic nicely bridges two distinct but intriguing fields: electron hydrodynamics and magnetoplasmonics. Overall, I consider the work suitable for publication in Nature Communications. However, I have several technical points that I would like the authors to address before I can recommend acceptance:

1. The authors mention that the electron temperature rises well above 1 K under microwave illumination. Have the authors performed a systematic study of the point-contact resistance as a function of lattice temperature? Such data would allow them to calibrate the effective electron temperature under microwave excitation. It would also be helpful to verify that the zero-field temperature dependence of the point-contact resistance is consistent with previous reports in the literature. Importantly, from such measurements, the authors could potentially estimate electron viscosity or the scattering length as a function of temperature. This would provide an independent check on the values extracted from the magnetic-field dependence of the microwave photoconductivity and significantly strengthen the conclusions.

2. The discussion focuses on the influence of magnetic field on the viscous length. However, have the authors considered the possible role of Hall viscosity in their measurements and analysis? If Hall viscosity is negligible in their geometry or frequency regime, the authors should explicitly state why it does not influence the observed behavior.

Version 1:

Reviewer comments:

Reviewer #1

(Remarks to the Author)
The authors improved the presentation and reasonably answered my technical questions. Therefore, I recommend the publication of the manuscript. Only one technical point to address: The authors explain that $P_a \sim T_e^2$ is due to electron-electron dominated cooling. I am not sure this is true - e-e collisions redistribute the energy and equilibrate the electronic system. Cooling happens due to thermal diffusion to leads or emission of phonons. Can the authors explain and provide a reference in the final manuscript on how and why e-e collisions cam lead to T_e^2 term in the energy balance equation. I understand that e-e collision rate is proportional to T_e^2 but cooling is a different process.

Reviewer #2

(Remarks to the Author)
I thank the authors for their detailed replies to my previous questions and concerns. Most of my comments below are largely about suggested improvements to the presentation.

5) I do agree with the authors that you can see signatures of l_{ee} even in the ballistic regime $l_{ee} >> W$, but I think one should not call transport in this regime hydrodynamic. If G_{PC} is 1.5 e^2/h it probably would be best to emphasize that one is likely not in a hydrodynamic regime but may nevertheless be able to extract signatures of l_{ee} following Ref. [24]. (Admittedly it is a little surprising if the kinetic theory predictions of Ref. [24] hold on such tiny scales but the data do speak for themselves.)

7) I think the authors misunderstood my concern here. If the electrons are being locally heated, then even within linear response (namely at 17 Hz) I would expect to have seen some thermoelectric effects. Based on the discussion on page 9 however, it appears the authors believe that the electron temperature varies drastically across the device, potentially taking one beyond the linear response regime. It may be the case that the electron density is sufficiently high that even relatively large thermal gradients are still negligible compared to viscous effects in the Navier-Stokes equations but it may be useful for the authors to at least acknowledge the issue further in the main text. There are also admittedly heating effects that are DC that I could understand as arising from dissipative heating, and I agree with the authors that their lock-in technique may filter that out.

8) I would still think that it makes more sense to fit to a model with exponent 0.5 if this is predicted by a simple theory of e.g. Joule heating. But this is a minor point so if the authors prefer to keep it as is I am OK with that. However my main concern in this point was about the way the electronic temperature is measured. I think if the authors are using the dependence of conductance on power of irradiation, it is hard to justify without assuming the validity of the hydrodynamic model. Perhaps one can say the following: we detect W^2 dependence in conductance consistent with hydrodynamics, and as such we use the theoretical prediction for electronic viscosity to estimate temperature. Of course the authors are not deep in the hydrodynamic regime so there may not be a clean closed-form formula to extract viscosity with, especially in a finite magnetic field. I think at a minimum a clearer acknowledgement of a potential limitation of the analysis is warranted.

Reviewer #3

(Remarks to the Author)
The authors have thoroughly addressed my questions and provided further analysis supporting their claims that strengthen

the quality and understanding of the manuscript. For this reason I can happily recommend the paper for publication

Version 2:

Reviewer comments:

Reviewer #1

(Remarks to the Author)
I support the publication. All questions have been answered.

Reviewer #2

(Remarks to the Author)
I am happy with the revisions and encourage publication.

Open Access This Peer Review File is licensed under a Creative Commons Attribution 4.0 International License, which permits use, sharing, adaptation, distribution and reproduction in any medium or format, as long as you give appropriate credit to the original author(s) and the source, provide a link to the Creative Commons license, and indicate if changes were made.
In cases where reviewers are anonymous, credit should be given to 'Anonymous Referee' and the source.
The images or other third party material in this Peer Review File are included in the article's Creative Commons license, unless indicated otherwise in a credit line to the material. If material is not included in the article's Creative Commons license and your intended use is not permitted by statutory regulation or exceeds the permitted use, you will need to obtain permission directly from the copyright holder.
To view a copy of this license, visit https://creativecommons.org/licenses/by/4.0/

We have carefully revised the manuscript, NCOMMS-25-58698A, in response to all reviewers' suggestions, with changes highlighted in red for ease of reference. Here is a summary of the major revisions made:

1. In the **Introduction** section, we removed redundant discussions on microwave-induced resistance oscillation and Kohn's theorem, and added a new paragraph on the mechanism of EMP-induced superballistic transport (page 4).
2. We substantially revised Fig. 1 and incorporated new measurement results on superballistic transport at zero magnetic field without microwave irradiation, as a function of temperature (pages 5-6), before introducing results under microwave irradiation.
3. We reorganized the presentation of our main results. After introducing hydrodynamic results and EMP physics, we first elaborate on the detailed mechanism of EMP-induced superballistic transport, followed by discussions on magnetic-field suppression, size dependence of superballistic transport, and the power-law relationship between electron temperature and microwave power. Finally, we present numerical simulations and length scales of hydrodynamic transport.
4. In the **Methods** section, we added a new subsection titled "**Local Heating vs. Global Thermalization**".
5. We included a discussion on Hall viscosity in Supplemental Material Section V.
6. We have carefully polished the manuscript to eliminate vague phrasing and redundant discussions of the same physical concepts.

We believe the revised version has been significantly improved thanks to their insightful recommendations, and we hope it is now suitable for publication. Below, we provide detailed responses to each reviewer's comments.

<u>Reviewer #1:</u>

The manuscript reports experiments on superballistic flow in high-mobility GaAs/AlGaAs point contacts under microwave irradiation, interpreted as arising from constructive interference of edge magnetoplasmons (EMPs) that locally heat electrons and drive them into the hydrodynamic regime. The topic is timely, and the underlying physics is interesting: the connection between EMP interference and viscous transport is actually exciting. However, in its current form the paper is very difficult to follow. The presentation is clouded by filler language, unnecessary detours into tangential topics, and figures that do not clearly emphasize the central message. I do recommend the publication but revision is required to make the work accessible to the broader readership of Nature Communications. Furthermore, the authors need to address the following questions.

Reply: We sincerely thank the reviewer for their thoughtful and constructive feedback on our manuscript. We agree that the presentation of the manuscript in its initial form could be improved for clarity and accessibility. In response, we have thoroughly revised the text

and figures to enhance the logical flow, remove tangential discussions, and emphasize the central message more effectively. Below, we address each of the reviewer's specific questions point by point.

Technical questions:

1) *Why not show point contact conductance vs regular temperature (by raising the bath T), or at least vs DC bias (to heat electrons)? This would establish the expected non-monotonic dependence (ballistic → superballistic → phonon) without relying only on microwaves first. This would be a proof that hydro is there in the first place without EMP. Then the MW power dependence could be compared to this benchmark.*

Reply: We thank the reviewer for this constructive suggestion. In our initial approach, we focused directly on the heating effect of interfering EMPs. The primary goal was to demonstrate a novel activation mechanism for the hydrodynamic regime, distinct from conventional global heating by bath temperature. Therefore, we presented the microwave (MW) power dependence as the central result. We acknowledge that in doing so, we did not explicitly pre-establish the baseline hydrodynamic behavior expected from global heating, which could make the argument less direct for some readers.

We fully agree with the reviewer that providing the suggested benchmark will make the paper more complete and persuasive. We have revised the manuscript as follows. We include new experimental data showing the non-monotonic point contact (PC) conductance as a function of bath temperature in Fig. 1. This data clearly shows the characteristic non-monotonic trend: conductance first increases (signature of superballistic flow due to enhanced hydrodynamic viscosity) as electrons are heated, reaches a maximum, and then decreases at higher bias due to phonon scattering (See the new Fig. 1 and related paragraphs on page 6).

2) *The authors assume that microwaves heat only the electron fluid in the constriction, but all absorbed energy must ultimately dissipate to contacts or phonons. How do they ensure that lattice temperature does not rise? What is the cooling power of the cryostat, and how do they know the lattice is thermalized to the bath? Can they provide evidence (e.g. monitoring bulk resistance or thermometer data) that the MW power does not heat the sample globally?*

Reply: We agree that it is essential to rule out global lattice heating by MWs as a trivial explanation for our observations. To address this concern, we have performed control experiments and analyses, which is clearly presented in the revised manuscript. In Methods (page 21), we clarify that "The most direct evidence comes from monitoring the SdH oscillations in the bulk resistance. Under strong MW irradiation (1000 μW), we still observe pronounced SdH oscillations at low magnetic fields of 2.4-2.8 kG (Fig. 7a). In contrast, these oscillations are known to be suppressed at temperatures above 1 K (Fig. 7b). The persistence of SdH oscillations under irradiation indicates that the global electron and lattice temperatures remain well below 1 K. Although some local dissipation is expected, these results confirm that the applied MW power does not cause substantial global heating

of the 2DEG. Fig. 7c shows the electron temperature in the 2DEG under MW irradiation, determined using the method described earlier."

Our experiments are conducted in a ^{3}He refrigerator with a base temperature of 300 mK and a cooling power exceeding 1 mW at 0.5 K - significantly exceeding the attenuated microwave power reaching the sample chamber. Note that the absorbed MW power by the insert chamber is much lower than P_{MW}. The sample is immersed in liquid ^{3}He, ensuring excellent thermal contact with the bath. Throughout the measurements, the rise in the liquid ^{3}He temperature under MW irradiation is below 0.02 K.

In summary, through direct experimental measurements and system characteristics, we provide strong evidence that MW irradiation does not raise the global lattice temperature above 1 K. The observed conductance enhancement is therefore not an artifact of overall sample warming but is consistent with localized electron heating driven by EMP interference within the PC. In the revised manuscript, we also add a dedicated paragraph summarizing these control experiments. The added section (page 10) states: "To rule out global heating, we monitored Shubnikov–de Haas (SdH) oscillations in the bulk 2DEG under MW irradiation. Their persistence at low magnetic fields confirms that the global electron temperature stays well below 1 K (see Methods and Fig. 7), a condition ensured by the cryostat's cooling capacity and effective thermal anchoring."

3) *The use of logarithmic dBm scale is standard in the microwave community but not intuitive for a general journal audience. Please provide plots in linear power units (mW) in addition to dBm, or at least a conversion.*

Reply: We thank the reviewer for this helpful suggestion to improve the accessibility of our figures for a broad audience. In the revised manuscript, we have provided plots in linear power units (μW) in addition to dBm.

4) *The authors state that the observed scaling of electron temperature with microwave power (T_e~P^0.6) is a signature of phonon cooling. Can the authors justify why they associate the measured exponent of 0.6 with phonon cooling?*

Reply: Thanks to the reviewers' question, we can interpret the exponent 0.6 in an appropriate way. The measured exponent of 0.6 is not associated with phonon cooling, but is related to electron-electron coupling. We revised the manuscript to interpret the measured exponent on page 13, "At thermal equilibrium, the absorbed MW power P_a is balanced by the power P_{tr} transferred from the PC region to the cold bath. This heat flow typically follows a power-law relation: $P_a = P_{tr} = \Sigma V\left(T_e^{\gamma} - T_b^{\gamma}\right)$, where T_b is the bath temperature, Σ is the electron-electron coupling constant, and V is the effective volume of the heated region. Under significant MW heating ($T_e \gg T_b$), the term T_b^{γ} is negligible. For electron-electron scattering (the bath is the cold bulk 2DEG), where $\gamma = 2$, this simplifies to $P_a \propto T_e^2$, yielding $T_e \propto P_a^{0.5}$. However, if electron-phonon scattering (the bath is sample lattice) were the dominant relaxation channel, the exponent would deviate from 0.5. In the electron-phonon coupling process, $P_a \propto T_e^5$, which would lead to a much weaker power dependence, $T_e \propto P_a^{0.2}$.".

***5)** Magnetic field affects the hydrodynamic flow via Hall viscosity [Science 364, 162-165, 2019] which can also affect the electromagnetic response of 2D fluids [Phys. Rev. B 94, 125427]. Can the authors comment whether this can affect their data or whether any signature of Hall viscosity can be found there.*

Reply: After careful consideration, we believe that the signatures of Hall viscosity are not prominent in our current experimental data. Hall viscosity is hard to measure because it does not directly contribute to standard linear-response transport coefficients like resistance. Experiments can probe secondary effects such as boundary effects, a measurable correction to the resistance at the edges near current injection point (*Science* 364, 162-165 (2019)).

An interesting point is that Hall viscosity effect happens at both of the two ends of a PC. However, there exists a cancellation problem. In a symmetric PC, Hall viscosity generates opposing electrical signatures on the upstream and downstream edges. In a simple two-terminal voltage measurement across the PC, these opposing signals would **cancel out**, resulting in a very weak net effect. This observation is further supported by the R_L traces, which exhibit minimal asymmetry in response to the magnetic field (Supplemental Material (SM) Fig. S5). If there would be some geometric asymmetry of the PCs, extremely weak Hall viscosity signals could be detected theoretically. Moreover, in our high-mobility GaAs/AlGaAs heterostructure at the magnetic fields used (on the order of 0.05-0.1T), the ratio of Hall viscosity to shear viscosity is estimated to be relatively small. Therefore, its contribution to the overall conductance is likely masked by the much stronger signal from viscosity effects.

In summary, while we appreciate the potential implications of Hall viscosity, our analysis indicates that the primary phenomenon reported here - the large enhancement of conductance - is driven by the hydrodynamic transport. We have revised the manuscript to include a discussion on this point in the new SM Section V.

Other comments:

***1)** The manuscript suffers from frequent use of vague phrases such as "natural and intriguing effects. These add little substance and obscure the actual results.*

Reply: We thank the reviewer for this constructive feedback. We have meticulously removed all such language throughout the text and replaced it with precise, scientific descriptions of our results and their implications. We believe the manuscript is now substantially improved owing to the reviewer's guidance.

***2)** Figures are dense and not always intuitive. For example, Fig. 2d does contain the superballistic signature, but this is only visible at the lowest MW powers. The key message (ballistic → superballistic → phonon scattering) could be emphasized far more clearly with schematic cartoons or by replotting data to highlight dips vs baseline. The paper is also hard to follow because the same physics is discussed repeatedly in slightly different ways. A tighter structure is needed.*

Reply: We have carefully considered the reviewer's main points regarding figure presentation and manuscript structure, and we will implement the following comprehensive revisions:

(i) Regarding figure clarity: We introduce the superballistic transport without MW irradiation in Fig. 1. For Fig. 2, we highlight the superballistic signature by filling the region with superballistic characteristics, and explicitly emphasize the "dips" relative to a clearly defined baseline to make the evolution of the transport regime unambiguous. We also add a new panel of Fig. 3a as a schematic diagram illustrating the effect of MW irradiation.

(ii) Regarding the manuscript's structure and repetitive discussion: We have undertaken a major revision of the manuscript. The results are presented in a more linear fashion, ensuring each section introduces a distinct concept that builds upon the previous one, leading clearly to our main conclusion. We have consolidated the discussion points to avoid repetition. Each physical mechanism is discussed thoroughly, but only once, in its most appropriate context.

We hope that these revisions will address the reviewer's concerns directly and result in a much stronger and more accessible paper. We thank the reviewer for their invaluable insights, which are crucial for enhancing the quality of our work.

3) *The introduction and background cite the authors' previous works intensively, including several MIRO studies that are not directly related to hydrodynamics. MIRO physics is a single-particle, impurity-scattering phenomenon and does not establish viscous flow. At best, MIRO shows that MWs couple to electrons in GaAs. In this paper, the relevant physics is instead EMP-driven heating of constrictions. The narrative should avoid conflating MIRO with hydrodynamics, as this distracts from the real novelty.*

Reply: We have comprehensively revised the manuscript to address this point directly and effectively. The discussion of MIRO is significantly reduced in the new manuscript. We hope that these revisions will eliminate the distraction, and ensure that the core contribution of the paper is presented with the clarity and emphasis it deserves.

Reviewer #2:

The authors do a set of interesting experiments to look for superballistic conductance in irradiated 2DEGs. The irradiation is supposed to enhance the momentum-conserving scattering rate in a way that was not clear to me but the authors have cited away to other papers. Assuming that this method indeed works, then what the authors propose is potentially intriguing. However, I found the paper to be not well-written, especially to non-technical experts in the field. Some of the comments below are just basic questions that should be more carefully explained in the main text, while others are more basic questions

I have about the authors' setup and the reliability of the results. I think these issues need to be addressed; at present I could not recommend the paper for publication.

Reply: We sincerely appreciate the reviewer acknowledging the potential intrigue of our work. We have undertaken a major revision of the manuscript to address all the points the reviewer raised. The revised manuscript is now substantially more accessible and robust. We sincerely hope that the extensive revisions we have made in response to the reviewer's points will make our manuscript suitable for publication. We provide a point-by-point response to the reviewer's general comments below.

***1)** Previous authors have used irradiation to try and add residual resistivity to a sample, i.e. add impurities. It seems however like this is not what the authors are doing here, but they do not really explain what the nature of the irradiation is. Are they trying to damage the sample or is the radiation turned on and off to try and stimulate an additional scattering mechanism on a fixed sample (i.e. that should not be damaged by the radiation).*

Reply: In our experiment, we are not attempting to damage the sample. The MW irradiation we use operates at low power levels and low frequencies that are incapable of creating defects in the GaAs/AlGaAs heterostructure. MWs are used to dynamically manipulate the electron scattering rates within a high-mobility device. We have added explicit text in the Methods section that clearly states: "The applied MW irradiation was non-destructive and was cycled on and off during measurements. Its sole purpose was to excite the electron system via EMPs, resulting in electron heating. This process is fully reversible and does not alter the intrinsic disorder of the sample."

***2)** The explanation based on Kohn's Theorem is unclear. Breaking translational symmetry would also presumably allow for momentum-non-conserving collisions. Kohn's Theorem can also be violated by breaking Galilean invariance of a parabolic dispersion relation in a 2DEG, e.g. or by considering the exchange of momentum between phonons and electrons (which together do not have Galilean invariance).*

Reply: We thank the reviewer for this insightful comment regarding Kohn's theorem. The reviewer is correct that breaking translational symmetry would also allow for momentum-non-conserving collisions and Kohn's theorem can also be violated by considering the exchange of momentum between electrons and phonons.

After careful consideration, we have decided to delete the discussion about Kohn's Theorem, which is not the main topic of this manuscript, since it could be unproper and misleading for the discussion of EMP-induced superballistic transport. The revised manuscript in **Introduction** section explains the physics of EMP-induced superballistic transport in a better way (page 4).

***3)** I didn't find Equation #1 to be very illustrative (it's also labeled somewhat awkwardly). Is it actually true that the dispersion relation depends on q rather than |q|?*

Reply: We thank the reviewer for pointing out the issue with Equation #1. We have deleted the equation label since it is not referenced except in the same paragraph.

Regarding the "q vs. |q|" question, the relation should indeed depend on the magnitude |q|, not the vector q. We have corrected this to properly reflect the dependence on the wavevector magnitude.

To make it more illustrative, we have pointed out that "The wave vector magnitude $|q|$, which scales linearly with magnetic field B, is determined from the EMP dispersion relation (Eq. (6) - (7)) [45]: $|q| = \epsilon_0(\epsilon_r + 1)m^*\omega(0.80\omega_c + 1.22\omega)/ne^2$". The main function of this equation is to illustrate the EMP nature of the resistance oscillations, by showing that they are theoretically periodic with $|B|$ and through the periodicity we can measure the propagation length of EMPs.

4) *To me it is confusing to see a negative P_MW, I think the authors are really reporting \log P_MW and reporting the answer in decibels.*

Reply: The reviewer is totally correct that "*the authors are really reporting \log P_MW and reporting the answer in decibels*". For example, 0dBm is equal to 1mW and -10dBm is equal to 0.1mW. We have just realized that the use of logarithmic scale (dBm) is standard in the microwave community but not intuitive for a general journal audience. In the revised manuscript, we have provided plots in linear power units (mW) in addition to dBm.

5) *What is the value of G_PC in the data shown in Figure 2d? How many multiples of the fundamental unit of conductance is it? This seems to have a very interesting interpretation from the perspective of hydrodynamics, i.e. you might think that N_PC must be large compared to 1 for viscous fluid flow to be relevant (since hydrodynamics depends on multi-particle scattering).*

Reply: We thank the reviewer for pointing out this subtlety. The value of G_{PC} in Figure 2d is about $1.5e^2/h$, indicating that N_{PC} is slightly smaller than 1. We acknowledge that hydrodynamic transport dominates when $N_{PC} \gg 1$, but this fact is not contradictory to our findings in the data of Figure 2d. Although this figure shows a point contact (PC) in the nearly ballistic regime, it can share some weak superballistic characteristics when the electron temperature is high enough. In Ref. [24], it claims that superballistic transport does not require the extreme hydrodynamic regime ($l_{ee} \ll W$) and weak viscous effects can manifest even near the ballistic regime ($l_{ee} \gg W$), which is exactly the case shown in this figure. Additionally, the relative change of the R_L under irradiation is almost negligible (less than 2%) owing to the ballistic dominated regime. In our manuscript, Figure 2 is plot to show EMP physics without highlighting the hydrodynamic transport and Figure 3 shows the data of hydrodynamic transport induced by EMPs.

In the revised manuscript, we discussed the specific data shown in Figure 2d and pointed out its near-ballistic nature (page 9): "The superballistic signature in Fig. 2d appears relatively subtle due to the limited number of conduction channels and near-ballistic character of this PC. More pronounced examples will be presented later using other PCs.".

6) *The formula #4 is explained in an awkward way. If the magnetic field is extremely small then the set of inequalities above the equation would not hold, since \beta would be extremely small. So actually this formula seems to apply in fairly large magnetic fields.*

Reply: We appreciate the reviewer pointing out the limitation of Formula #4. The reviewer is correct that the inequality preceding it inherently requires a sufficiently large magnetic field to be valid. We have pointed out explicitly that the formula applies under the condition of $k_B^2 T_e^2 / E_F^2 \ll \beta \ll 1$. Since E_F of our samples is extremely high, around $110K$, much higher than the electron temperature in the superballistic regime, we can estimate that Formula #4 can be used when magnetic field B is larger than $0.2kG$ for the PCs in our manuscript. We see that the lower limit of the magnetic field is small enough, indicating that for the magnetic field with constructive interference of EMPs (normally larger than $0.4kG$), the condition for Formula #4 is not violated.

For clarity, we denote the condition where Formula #4 works well in the main text (page 11), "Within the regime $k_B^2 T_e^2 / E_F^2 \ll \beta \ll 1$ (corresponding to magnetic fields of approximately $0.2\ kG - 2\ kG$)".

7) *Why can thermoelectric effects be ignored? If the electron system is drastically heating up then this would change the character of hydrodynamic flow. I didn't understand how the lock-in technique filters out these signals. I would think that thermoelectric transport enters the theoretical modeling of the experiment by introducing a new set of boundary conditions on temperature and/or heat current at the contacts. Depending on what these boundary conditions are can have big consequences on the resulting conductivity in hydrodynamics, as was emphasized in the literature on the Wiedemann-Franz law that the authors cite.*

Reply: The reviewer is correct that a heated electron system can generate thermoelectric voltages (e.g., Seebeck effect). However, our lock-in amplifier measurement scheme is specifically designed to reject such signals. The fundamental reason is that thermoelectric effects, such as the Seebeck voltage, are primarily DC phenomena. Our measurement applies a small AC excitation current at a low frequency $f = 17Hz$ to measure the conductance. The lock-in amplifier detects the voltage response at this specific frequency f. A steady-state thermal gradient would produce a DC voltage offset, which is rejected by the lock-in. We have added an explanation in the Methods section (page 18) which clarifies: "The linear conductance was measured using a lock-in technique, which detects the in-phase voltage response to an AC current. This approach effectively filters out DC thermoelectric contributions."

If positive photoconductance of PCs is dominated by the remaining thermoelectric signals, its sign will turn negative when we switch the current to the opposite orientation. This is because thermal voltage does not reverse its direction while the current does (thermal voltage is decided by MW irradiation). This does not happen in our samples. This result directly rules out the possibility of dominant thermoelectric signals. We revised the manuscript to add such comments in the Supplemental Material (SM) Section II.

We acknowledge that a complete hydrodynamic model includes thermoelectric couplings could be comprehensive. However, for the specific transport quantity we measure - the conductance at $f = 17Hz$ - the dominant effect of the MW-induced heating is to elevate the local electron temperature, which primarily enhances the electron-electron scattering rate τ_{ee}, driving the system into the hydrodynamic regime. In our system, the primary role of the EMP-driven heating is to reduce τ_{ee}, thereby modifying the effective viscosity. This is the core mechanism we intend to capture in our simplified model.

8) *I was not sure about how the authors estimate the electron temperature, which they say can be very large from the irradiation. The fitting of temperature seems to me to come from the hydrodynamic model itself, but is that not the model the authors are trying to confirm with their analysis? While I could still study the W^2 dependence of conductance it seems that this fitting approach may limit the extent to which the authors can verify another aspect of the hydrodynamic model? Also, the 0.6 exponent relating T and P seems ad hoc to me and not likely to be fundamental.*

Reply: We thank the referee for this critical question regarding the estimation of electron temperature and its potential to introduce circularity into our argument. The referee has correctly identified a key challenge in this field: directly and independently measuring the local electron temperature in a constriction under MW irradiation is notoriously difficult. We acknowledge the potential concern and have taken the following steps to strengthen our claims. The key is that we use conclusion of our hydrodynamic model to derive the relation between T_e and P_a, which shows a universal rule for PCs we used.

First, our argument is not based solely on this self-consistent fitting procedure. We have characterized the superballistic behavior of our PCs (page 6), as shown in the new Fig. 1, and these results are consistent with prior work. The most direct evidence supporting hydrodynamic transport is the observed non-monotonic dependence of conductance on temperature. This non-monotonicity serves as a qualitative hallmark of the interplay between viscous and diffusive transport mechanisms.

Furthermore, we have measured the resistance R_L at various bath temperatures to provide a qualitative comparison with the data obtained under MW irradiation (SM Fig. S5). While the trends are similar, it is important to note that the results from varying the bath temperature cannot be directly used to calibrate the electron temperature under MW irradiation. The key distinction lies in the heating profiles: under MW irradiation, Joule heating is highly localized, raising the temperature in the PC region to approximately 10 K while the 2DEG temperature remains below 1 K. In contrast, when the thermal bath temperature is increased, both the 2DEG and the PC region are heated uniformly. This fundamental difference can lead to significant disparities that are challenging to quantify T_e under MW irradiation. We added **Local heating vs global thermalization** Section in Methods to explicitly clarify this point.

Regarding the the 0.6 exponent (to be exact, the number is 0.56) relating T_e and P_a, we can interpret the exponent 0.56 in an appropriate way. We revised the manuscript to interpret the measured exponent on page 13, "At thermal equilibrium, the absorbed MW

power P_a is balanced by the power P_{tr} transferred from the PC region to the cold bath. This heat flow typically follows a power-law relation: $P_a = P_{tr} = \Sigma V\left(T_e^{\gamma} - T_b^{\gamma}\right)$, where T_b is the bath temperature, Σ is the electron-electron coupling constant, and V is the effective volume of the heated region. Under significant MW heating ($T_e \gg T_b$), the term T_b^{γ} is negligible. For electron-electron scattering (the bath is the cold bulk 2DEG), where $\gamma = 2$, this simplifies to $P_a \propto T_e^2$, yielding $T_e \propto P_a^{0.5}$. However, if electron-phonon scattering (the bath is sample lattice) were the dominant relaxation channel, the exponent would deviate from 0.5. In the electron-phonon coupling process, $P_a \propto T_e^5$, which would lead to a much weaker power dependence, $T_e \propto P_a^{0.2}$ …This exponent is close to the theoretical value of $\gamma = 2$, yielding $1/\gamma = 0.5$, which is a hallmark of energy relaxation dominated by electron-electron coupling. The observed scaling thus provides strong evidence that the thermal relaxation in our samples is mediated primarily by electron-electron interactions.”.

9) *I had a basic physics question about the authors' setup as well. What exactly is the role of the EMP doing here, is it providing additional degrees of freedom to the scatter the electrons off that are flowing far away from the edge? In a narrow PC, how do I cleanly disentangle transport from EMP vs. transport from the hydrodynamic electrons?*

Reply: We illustrate here the main mechanism of MW irradiation in our devices (page 4). MW irradiation is absorbed by the 2DEG, resulting in the excitation of EMPs at the boundaries of the sample. This absorbed energy does not directly create more collisions or provide additional degrees of freedom to scatter the electrons. Instead, it heats up the electrons in the PC region (due to large photocurrent density), raising the effective electron temperature T_e significantly above the lattice temperature. Therefore, the MW irradiation indirectly enhances the momentum-conserving electron-electron scattering rate $1/\tau_{ee}$ via heating. When $1/\tau_{ee}$ is comparable with other scattering timescale, the system reaches the hydrodynamic regime. This logical chain is now clearly laid out in the introduction part for intuitive understanding, ensuring that the core premise of the paper is no longer “cited away” but explained in full.

The contributions from EMPs and bulk hydrodynamic electron flow can be clearly distinguished based on the vastly different frequencies of the driving signals involved. The EMPs are excited by the external MW irradiation, which operates at a high frequency (approximately 70 GHz). These plasmon waves are generated near the metallic contacts, propagate along the sample boundaries, and constructively interfere within the PC region. Crucially, our electrical transport measurements are performed using a current source at a very low frequency of 17 Hz. The measured conductance, which exhibits the non-monotonic behavior characteristic of hydrodynamic flow, is therefore the system’s low-frequency response to the high-frequency excitation provided by the EMPs. The EMPs act as a high-frequency stimulus that heats the electron fluid, while the low-frequency measurement probes the resulting steady-state transport properties of the PCs. This separation of scales - between the high-frequency driving mechanism (EMPs at ~70 GHz) and the low-frequency measurement (~17 Hz) - allows us to unambiguously attribute the observed non-monotonic conductance to the induced hydrodynamic behavior, rather than to a direct high-frequency transport signal from the EMPs themselves.

Reviewer #3:

The authors present an experiment on point contacts of two-dimensional electron gases under microwave illumination and magnetic field. They study the microwave photoconductivity of point contacts as a function of magnetic field and observe oscillatory structures. The results are explained in terms of selective heating via edge magnetoplasmons, which push the system in and out of the hydrodynamic regime through enhanced electron–electron collisions. I find the manuscript to be technically sound, and the results appear reliable. The topic nicely bridges two distinct but intriguing fields: electron hydrodynamics and magnetoplasmonics. Overall, I consider the work suitable for publication in Nature Communications. However, I have several technical points that I would like the authors to address before I can recommend acceptance:

Reply: We thank the reviewer for their positive and constructive assessment of our manuscript, and for their encouraging comments regarding its suitability for publication in *Nature Communications*. We have carefully considered all their technical points and have revised the manuscript accordingly to address them thoroughly. Below, we provide a point-by-point response, and we believe these revisions have significantly strengthened the paper.

1) *The authors mention that the electron temperature rises well above 1 K under microwave illumination. Have the authors performed a systematic study of the point-contact resistance as a function of lattice temperature? Such data would allow them to calibrate the effective electron temperature under microwave excitation. It would also be helpful to verify that the zero-field temperature dependence of the point-contact resistance is consistent with previous reports in the literature. Importantly, from such measurements, the authors could potentially estimate electron viscosity or the scattering length as a function of temperature. This would provide an independent check on the values extracted from the magnetic-field dependence of the microwave photoconductivity and significantly strengthen the conclusions.*

Reply: As suggested, we have performed measurements of the point-contact (PC) resistance as a function of lattice temperature in the absence of microwave (MW) irradiation. This data, now included as the new Fig. 1, provides an independent check. The zero-field temperature dependence we measured (Fig. 1) is fully consistent with previous reports on superballistic PCs (Ref. [24]). We have also used the temperature-dependent resistance data to estimate the electron-electron scattering length l_{ee} as a function of temperature. The extracted values of $l_{ee}(T)$ are now presented in the new Fig. 1d and discussed on page 6. Importantly, these independently derived values are in agreement with the range of l_{ee} values inferred from the MW photoconductivity.

We have measured the resistance R_L at various bath temperatures to provide a qualitative comparison with the data obtained under MW irradiation (Supplemental Material (SM) Fig. S5). While the trends are similar, it is important to note that the results from varying the

bath temperature cannot be directly used to calibrate the electron temperature under MW irradiation. The reason is listed in Methods on page 22.

2) *The discussion focuses on the influence of magnetic field on the viscous length. However, have the authors considered the possible role of Hall viscosity in their measurements and analysis? If Hall viscosity is negligible in their geometry or frequency regime, the authors should explicitly state why it does not influence the observed behavior.*

Reply: We thank the reviewer for raising this insightful point regarding the potential role of Hall viscosity in magneto-hydrodynamic transport. We agree that Hall viscosity, as a dissipationless component of the viscosity tensor arising in magnetic fields, can in principle modify the electromagnetic response and flow profiles of two-dimensional electron fluids.

We believe that the signatures of Hall viscosity are not prominent in our experimental data. We have explicitly stated the reason why it does not influence the observed results in SM Section V. In short, Hall viscosity generates opposing electrical signatures on the upstream and downstream edges. These opposing signals would cancel out, resulting in a very weak net effect.

We have revised the manuscript, NCOMMS-25-58698A, in response to all reviewers' suggestions, with changes highlighted in red for ease of reference. Here is a summary of the revisions made:

1. In the **Method** section, we add a new subsection called **Heat flow through point contacts** in order to introduce a model explaining the power-temperature relation.
2. We acknowledge certain limitations in our experimental methodology in the main text.

We believe the revised version has been improved thanks to the reviewers' insightful recommendations, and we hope it is now suitable for publication. Below, we provide detailed responses to each reviewer's comments.

<u>Reviewer #1:</u>

The authors improved the presentation and reasonably answered my technical questions. Therefore, I recommend the publication of the manuscript. Only one technical point to address: The authors explain that P_a~T_e^2 is due to electron-electron dominated cooling. I am not sure this is true - e-e collisions redistribute the energy and equilibrate the electronic system. Cooling happens due to thermal diffusion to leads or emission of phonons. Can the authors explain and provide a reference in the final manuscript on how and why e-e collisions can lead to T_e^2 term in the energy balance equation. I understand that e-e collision rate is proportional to T_e^2 but cooling is a different process.

Reply: We thank the reviewer for their positive feedback and recommendation for publication, as well as for their insightful technical comment regarding the interpretation of the $P_a \sim T_e^2$ scaling in relation to electron-electron (e-e) collision-dominated cooling.

We agree with the reviewer that the cooling mechanism warrants careful consideration. In our system, we find that attributing the T_e^2 scaling solely to e-e collisions may be problematic, as the cooling process is complex and requires further study. Therefore, in the revised manuscript, we have proposed and elaborated on an alternative scenario that also yields a T_e^2 dependence. While this model is not directly based on e-e collisions, it is well-established for point contacts (PCs) and involves electron-mediated heat relaxation, which we believe is more consistent with our experimental conditions.

Specifically, in the Methods section, we have added the following explanation (paraphrased here for clarity): "For narrow constrictions such as our PCs, the thermal conductance K in the near-ballistic transport regime scales linearly with electron temperature T_e. In the ballistic limit, $K = N_{\mathrm{PC}} \cdot \pi k_B^2 T_e / 3\hbar$ [51], consistent with the Wiedemann-Franz law. In our experiment, the PCs are heated by electromagnetic pulses (EMPs), creating a temperature gradient between the PC region and the bulk 2DEG. The resulting heat flow can be expressed as $P_{\mathrm{tr}} = K \nabla T_e \approx \pi k_B^2 N_{\mathrm{PC}} (T_e^2 - T_b^2) / 6\hbar l_{\mathrm{PC}}$ (the Peltier term cancels out). Here, the bulk 2DEG rather than the lattice acts as the dominant

cold bath for hot electrons in the PC, due to the relatively weak electron-phonon coupling at lattice temperatures around 0.3 K. This model naturally explains the observed quadratic dependence $P_a \propto T_e^2$, and consequently $T_e \propto P_a^{0.5}$. These results indicate the presence of a dominant electron-mediated heat relaxation channel for hot electrons within the PCs."

The key point is that the observed power-law relationship $T_e \propto P_{\mathrm{MW}}^{*0.56}$ reflects a heat relaxation process distinct from lattice cooling, which arises from the small size of the PCs and the weak electron-phonon coupling at our experimental temperatures. We believe that this PC cooling model provides a suitable and consistent explanation for our results. We also anticipate that future studies will investigate the thermal conductance of superballistic electron flow.

We hope this clarification addresses the reviewer's comment. If this is not the case, we believe this remains a minor point that does not affect the central conclusions of the article. We have incorporated the discussion into the revised manuscript and thank the reviewer again for raising this important point.

<u>Reviewer #2</u>:

I thank the authors for their detailed replies to my previous questions and concerns. Most of my comments below are largely about suggested improvements to the presentation.

Reply: We thank the reviewer for acknowledging our previous responses. We sincerely appreciate the additional feedback aimed at improving the presentation of the manuscript. We carefully incorporate the suggestions to enhance the clarity, structure, and overall quality of the paper.

5) *I do agree with the authors that you can see signatures of l_ee even in the ballistic regime l_ee >> W, but I think one should not call transport in this regime hydrodynamic. If G_{PC} is 1.5 e^2/h it probably would be best to emphasize that one is likely not in a hydrodynamic regime but may nevertheless be able to extract signatures of l_ee following Ref. [24]. (Admittedly it is a little surprising if the kinetic theory predictions of Ref. [24] hold on such tiny scales but the data do speak for themselves.)*

Reply: We absolutely agree with the reviewer's point regarding the terminology of "hydrodynamic" in the ballistic regime where $l_{ee} \gg W$. In the revised manuscript, we have emphasized that while signatures of l_{ee} can indeed be observed even in this regime, the transport should not be described as hydrodynamic (although with some characteristics). We note that the measured conductance of $G_{PC} \approx 1.5\, e^2/h$ supports that the regime is not hydrodynamic, while still being consistent with the model in Ref. [24]. In the revised manuscript, it reads "The superballistic signature in Fig. 2d appears relatively subtle due to the limited number of conduction channels and near-ballistic character of this PC (Although superballistic signature exists, the PC is not in the hydrodynamic regime).".

7) *I think the authors misunderstood my concern here. If the electrons are being locally heated, then even within linear response (namely at 17 Hz) I would expect to have seen some thermoelectric effects. Based on the discussion on page 9 however, it appears the authors believe that the electron temperature varies drastically across the device, potentially taking one beyond the linear response regime. It may be the case that the electron density is sufficiently high that even relatively large thermal gradients are still negligible compared to viscous effects in the Navier-Stokes equations but it may be useful for the authors to at least acknowledge the issue further in the main text. There are also admittedly heating effects that are DC that I could understand as arising from dissipative heating, and I agree with the authors that their lock-in technique may filter that out.*

Reply: We thank the reviewer for their detailed clarification and for raising this important point regarding thermoelectric effects. The reviewer is correct that local temperature gradients could in principle generate thermoelectric voltages. Although no direct evidence of the thermoelectric effect was found in our data, and the net thermoelectric voltage is canceled out by the symmetric measurement geometry, this effect may still have exerted some influence on the experiments.

In the revised manuscript, we have acknowledged this issue in the **Discussion** part, "However, we cannot rule out the possibility that thermoelectric effect may still have some influence in our experiments, given that thermal gradients compete with viscous effects in the governing Navier-Stokes equations.".

8) *I would still think that it makes more sense to fit to a model with exponent 0.5 if this is predicted by a simple theory of e.g. Joule heating. But this is a minor point so if the authors prefer to keep it as is I am OK with that. However my main concern in this point was about the way the electronic temperature is measured. I think if the authors are using the dependence of conductance on power of irradiation, it is hard to justify without assuming the validity of the hydrodynamic model. Perhaps one can say the following: we detect W^2 dependence in conductance consistent with hydrodynamics, and as such we use the theoretical prediction for electronic viscosity to estimate temperature. Of course the authors are not deep in the hydrodynamic regime so there may not be a clean closed-form formula to extract viscosity with, especially in a finite magnetic field. I think at a minimum a clearer acknowledgement of a potential limitation of the analysis is warranted.*

Reply: We fully agree that extracting temperature from the conductance's power dependence relies on assumptions tied to the hydrodynamic model. In the revised manuscript, we have included a clear statement acknowledging this as a potential limitation of our analysis, emphasizing that the temperature estimates are model-dependent and should be interpreted within the context of the hydrodynamic framework: "Our analysis is limited by the lack of a direct measurement for T_e, and therefore proceeds under the assumption of hydrodynamic model.".

We believe that this added clarification will provide a more transparent and balanced presentation of our methodology and its assumptions.

<u>Reviewer #3:</u>

The authors have thoroughly addressed my questions and provided further analysis supporting their claims that strengthen the quality and understanding of the manuscript. For this reason I can happily recommend the paper for publication.

Reply: We thank the reviewer for their positive feedback and for recommending our paper for publication. We sincerely appreciate the time and effort they dedicated to reviewing our work and for providing valuable insights that have helped improve its quality.
